# A 24-48 h fed *Amblyomma americanum* tick saliva immuno-proteome

**DOI:** 10.1186/1471-2164-15-518

**Published:** 2014-06-24

**Authors:** Željko M Radulović, Tae K Kim, Lindsay M Porter, Sing-Hoi Sze, Lauren Lewis, Albert Mulenga

**Affiliations:** Department of Entomology, AgriLife Research, Texas A & M University, 2475 TAMU, College Station, TX77843 USA; Department of Computer Sciences and Engineering, Texas A & M University, College Station, TX77843 USA; Department of Biochemistry & Biophysics, Texas A & M University, College Station, TX77843 USA

**Keywords:** *Amblyomma americanum*, Tick saliva proteins, Biopanning, Immuno-proteome

## Abstract

**Background:**

Multiple tick saliva proteins, the majority of which are unknown, confer tick resistance in repeatedly infested animals. The objective of this study was to identify the 24-48 h fed *Amblyomma americanum* tick saliva immuno-proteome. The 24-48 h tick-feeding phase is critical to tick parasitism as it precedes important events in tick biology, blood meal feeding and disease agent transmission. Fed male, 24 and 96 h fed female phage display cDNA expression libraries were biopanned using rabbit antibodies to 24 and 48 h fed *A. americanum* female tick saliva proteins. Biopanned immuno-cDNA libraries were subjected to next generation sequencing, *de novo* assembly, and bioinformatic analysis.

**Results:**

More than 800 transcripts that code for 24-48 h fed *A. americanum* immuno-proteins are described. Of the 895 immuno-proteins, 52% (464/895) were provisionally identified based on matches in GenBank. Of these, ~19% (86/464) show high level of identity to other tick hypothetical proteins, and the rest include putative proteases (serine, cysteine, leukotriene A-4 hydrolase, carboxypeptidases, and metalloproteases), protease inhibitors (serine and cysteine protease inhibitors, tick carboxypeptidase inhibitor), and transporters and/or ligand binding proteins (histamine binding/lipocalin, fatty acid binding, calreticulin, hemelipoprotein, IgG binding protein, ferritin, insulin-like growth factor binding proteins, and evasin). Others include enzymes (glutathione transferase, cytochrome oxidase, protein disulfide isomerase), ribosomal proteins, and those of miscellaneous functions (histamine release factor, selenoproteins, tetraspanin, defensin, heat shock proteins).

**Conclusions:**

Data here demonstrate that *A. americanum* secretes a complex cocktail of immunogenic tick saliva proteins during the first 24-48 h of feeding. Of significance, previously validated immunogenic tick saliva proteins including AV422 protein, calreticulin, histamine release factor, histamine binding/lipocalins, selenoproteins, and paramyosin were identified in this screen, supporting the specificity of the approach in this study. While descriptive, this study opens opportunities for in-depth tick feeding physiology studies.

**Electronic supplementary material:**

The online version of this article (doi:10.1186/1471-2164-15-518) contains supplementary material, which is available to authorized users.

## Background

Ticks are reservoirs and vectors of numerous animal and human pathogenic microorganisms, including bacteria, viruses, and protozoans. Although in terms of public health impact ticks are considered second to mosquitoes, they surpass any arthropod vector in terms of diversity of disease agents that they transmit and their impact on livestock production [1, 2]. For many years ticks and tick borne diseases were considered a veterinary problem, where economic losses run into several millions of US$ annually [3]. In Brazil alone, loses due to the cattle tick, *Rhipicephalus microplus* were estimated at 2 billion US$ annually [4]. However in recent years, the impact of human tick borne diseases in public health have been growing.

*Amblyomma americanum*, previously considered a nuisance, is among important tick species in public health [5]. This tick previously established in southeastern United States has now spread to the northeast [6, 7]. *A. americanum* has been reported as the most pre-dominant tick species found on humans in this part of the United States [8]. This species transmits several human tick borne disease agents including *Ehrlichia chaffeensis*, *Ehrlichia ewingii*, and *Francisella tularensis* [9–14]. *A. americanum* also transmits the causative agents of southern tick associated rash illness (STARI) [15, 16], *Ehrlichia ruminatium-*like Panola Mountain *Ehrlichia* (PME) [17, 18], and has also been linked to Heartland virus [19]. There is also evidence that *A. americanum* may transmit *Rickettsia amblyommii* to humans [20]. In veterinary health, *A. americanum* transmits *Theileria cervi* to deer [21], and *E. ewingii* to dogs [22]. There are reports of mortality in deer fawns that were attributed to a combination of heavy *A. americanum* infestation and *T. cervi* infections [23].

Although chemical acaricide based strategies represent the dominant prevention method against tick borne disease infections, the focus is moving to developing new, more efficient and environmentally friendly strategies [24]. One of the possible alternative strategies could be the production of anti-tick vaccines. This idea is not new, as it is known for more than 80 years that immunity to tick infestation could be induced by vaccination with a whole tick or salivary gland homogenates [25, 26]. Currently, the focus is on identification of efficacious tick protein antigens, which could be expressed as recombinant vaccine antigens [27–29]. Generally, there are two groups of these antigens. The first, so called “exposed antigens” includes tick proteins that are injected into the host during the tick feeding process. The second group of antigens, known as “concealed antigens”, refers to molecules which are not in direct contact with the host and usually do not induce an immunological response, such as tick gut components [30]. In our lab we are interested in “exposed antigens” and in the prospect of finding target anti-tick vaccine antigens, in which subsequent tick infestations of immunized animals will trigger an anamnestic (elevated) antibody response and serve as a “de facto” booster shot. In this way the need for manual administering of booster shots to the host will be eliminated.

Bioactive molecules in tick saliva play important roles in facilitating blood meal feeding and transmission of tick borne disease agents. The tick feeding style of lacerating host tissue and then sucking up blood that bleeds into the wounded area is expected to stimulate host defense responses aimed at stopping blood loss and initiating tissue repair responses. Expected host responses to tick feeding activity include vasoconstriction, platelet aggregation, fibrin clot formation, inflammation, and complement activation [31]. Studies to find tick saliva proteins that facilitate feeding were modeled after the expected host responses to tick feeding. In this way vasodilators [32–35], inhibitors of platelet aggregation [36–38], anti-coagulants [39–52], anti-inflammatory proteins [53, 54], and inhibitors of complement activation [55–59] were described in several tick species. Other studies have identified apparent pain blockers, a metallo dipeptidyl carboxypeptidase from saliva of *Ixodes scapularis* [60] and a thiol-activated metalloendopeptidase from saliva of *R. microplus* [61]. In a related study, Mulenga et al. [62] and Mulenga and Azad [63] described a functional histamine release factor (HRF) in tick saliva. The presence of HRF in tick saliva was considered counter-intuitive in that HRF is pro-inflammatory [64], and on the other hand, ticks should stop the inflammation response to feed successfully.

The idea of immunizing animals against tick feeding was prompted by observations that repeated tick infestation of animals conferred protective anti-tick immunity [65, 66]. In subsequent studies immune sera of tick resistant animals bound numerous protein bands from tick salivary gland protein extracts [67, 68], suggesting that numerous tick saliva proteins provoked anti-tick immunity in repeatedly infested animals. The objective of this study was to identify and characterize 24-48 h fed *A. americanum* tick saliva immunogenic protein coding cDNAs. We are interested in 24-48 h post attachment tick saliva proteins because this tick feeding stage precedes some of the most important facets of tick parasitism, blood meal feeding, transmission, and acquisition of tick borne disease agents.

## Methods

### Ticks

Unfed *A. americanum* ticks for this study were purchased from tick laboratories located at Texas A&M University and Oklahoma State University. In our lab, ticks were kept at favorable conditions (room temperature and > 85% relative humidity) and fed on New Zealand White Rabbits according to the animal use protocol #2011-189 approved by Texas A & M University IACUC to AM. Six male ticks were pre-fed for three days before placing female ticks to feed. To prevent ticks from entering the inner ear, they were restricted onto the top of the rabbit ear using an orthopedic stockinet containment cell adhered onto rabbit skin using the Kamar Adhesive (Kamar Products Inc., Zionsville, IN). Fed male ticks were collected from several feeding experiments, while female ticks were manually detached at 24 and 96 h post attachment and processed for RNA extraction as described below.

Following detachment, pieces of rabbit skin were manually cleaned off the tick mouthparts using soft tissue forceps. Subsequently ticks were washed in dietylpyrocarbonate (DEPC) treated water and dried on a paper towel. Pools of eight to ten ticks were chopped up using a sharp razor blade and homogenized in 1 mL TRIzol (Life Technologies, Carlsbad, CA, USA) and stored at −80°C until total RNA extraction.

### Messenger RNA extraction and cDNA preparation

Total RNA was extracted according to the detailed protocol provided by TRIzol reagent manufacturer (Life Technologies). Isolation of mRNA from prepared total RNA was done using Straight A’s™ mRNA Isolation System (Novagen, Madison, WI, USA). Total RNA was mixed with Magnetictight Oligo(dT) Particles (20 μg of particles per 1 μg of total RNA). After appropriate washes mRNA was eluted in 0.5 mL of nuclease free water by incubating at 60°C for 10 minutes. Subsequently mRNA was concentrated by mixing sample with 2 μL of Glycogen (10 mg/mL), 50 μL of 3 M sodium acetate, and 331 μL of 2-propanol, and centrifugation at 14000 × g for 5 minutes. The mRNA pellet was washed with 0.5 mL of 70% ethanol and dissolved in 25 μL of nuclease free water. Concentration of mRNA samples was determined by measuring absorbance at 260 nm using the DU 640B spectrophotometer (Beckman Coulter, Brea, CA, USA).

### Fed male, 24 and 96 h fed female *A. americanum*phage display expression cDNA libraries

Phage display cDNA expression libraries were constructed using T7Select OrientExpress cDNA cloning System (Novagen, Madison, WI, USA). A total of 4 μg of mRNA was used to synthesize cDNA using the OrientExpress™ Oligo(dT) cDNA Synthesis Kit (Novagen, Madison, WI, USA) according to instructions. Prior to proceeding with the rest of the protocol, success of cDNA synthesis was verified by PCR amplification of tick actin gene sequence using ^5′^GGACAGCTACGTGGGCGACGAGG^3′^ and ^5′^CGATTTCACGCTCAGCCGTGGTGG^3′^ primers, and MyTaq Red Mix (Bioline USA Inc., Taunton, MA, USA). Prepared cDNA was stored at −20°C. Following verification of cDNA synthesis, cDNA ends were modified by ligating *Eco*RI and *Hin*dIII sticky ends at the 5′ and 3′ ends. Subsequently modified cDNA was ligated to directional *Eco*RI/*Hin*dIII linkers. After appropriate treatment and fractionation to remove excess linkers, cDNA was ligated into T7Select vector arms using T4 ligase (Novagen, Madison, WI, USA). Following ligation the library was packaged using T7 packaging extracts previously thawed on ice and mixed by stirring with a pipet tip. The packaging reaction was incubated for 2 h at room temperature and was stopped by adding 270 μL of sterile M9TB medium with carbenicillin (final concentration 50 μg/mL). The packaging reaction was mixed by inverting with 20 μL of chloroform and stored at 4°C.

The phage titer in the packaging reaction was determined by plaque assay [69]. Serial dilutions of packaging reaction in M9TB medium with carbenicillin, ranging from 1:10^3^ to 1:10^6^, were prepared. Fresh culture of *Escherichia coli* BLT5403 strain (OD_600_ = 1) in M9TB medium with carbenicillin was used as host cells for T7 phages. From each dilution, 100 μL were mixed with 250 μL of host cells and 3 mL of molten top agarose, and plated on LB agar plates with carbenicillin (final concentration 50 μg/mL). Plates were incubated at 37°C for 4 h and formed plaques were counted. The phage titer was calculated according to the formula provided in the user manual. After determining the titer, the library was amplified using the plate lysate amplification protocol according to instructions in the user manual. Phage was diluted to 1 × 10^6^ per 10 mL of host cells (OD_600_ = 0.6-1.0). One mL of phages/host cells mixture was combined with 10 mL of molten top agarose and plated on 150 mm LB agar plates with carbenicillin. Plates were incubated at 37°C for 3–4 hours. When plaques on the plates became nearly confluent, each plate was incubated with 10 mL of phage extraction buffer (20 mM Tris–HCl pH 8.0, 100 mM NaCl, 6 mM MgSO_4_) overnight at 4°C. Phage elutes were collected, mixed with 0.5 mL of chloroform, and clarified by centrifugation at 3000 g for 5 minutes. The supernatant was mixed with 0.1 volume of sterile 80% glycerol and stored at −80°C. The titer of amplified libraries was determined using protocol described above.

### Verifying quality of phage display libraries

Following amplification, the quality of the amplified libraries was verified by checking the length of cloned cDNA sequences. 10–15 plaques per library were inoculated in 5 mL of host cells (fresh culture of BLT5403 strain in LB medium with cabenicillin, OD_600_ = 0.5-0.6). Culture was incubated at 37°C with shaking until lysis was observed. Lysate was clarified by centrifugation at 8000 g for 10 minutes. For phage extraction 1.25 mL of 20% PEG-8000/2.5 M NaCl were mixed with 5 mL of the lysate and incubated on ice at least 30 minutes. Phages were pelleted by centrifugation at 11000 g for 20 minutes, and then re-suspended in 100 μL of STE Buffer (10 mM Tris–HCl pH 8.0, 100 mM NaCl, 1 mM EDTA). The suspension was centrifuged at 14000 g for 10 minutes and the supernatant containing phages was transferred to a fresh tube. Phage extracts were used as templates for PCR amplification of cloned cDNA using the T7Select UP (^5′^GGAGCTGTCGTATTCCAGTC^3′^) and T7Select DOWN (^5′^AACCCCTCAAGACCCGTTTA^3′^) primers. PCR products were separated by electrophoresis on 2% agarose gel.

### Production of antibodies to fed male and 24 h female *A. americanum*tick saliva proteins

Production of antibodies to 48 h fed *A. americanum* tick saliva proteins was previously described [70]. The same protocol was used to produce antibodies to 24 h fed *A. americanum* tick saliva proteins. Rabbits were infested with 30 female ticks (15 per ear) every 24 h three times per week. This routine was repeated for four times after which antibody response to tick saliva proteins was verified. Blood was collected from rabbits approximately two weeks after the last round of tick feeding according to the protocol #2011-189 approved by Texas A & M University IACUC to AM. Rabbits were exsanguinated following humane standards by qualified veterinarians as approved by the Texas A & M University Comparative Medicine Program. Blood was left to clot overnight at 4°C to achieve maximum serum separation. Collected serum was stored at −80°C. Prior to tick infestations, rabbits were bled according to approved Texas A & M University protocols to collect pre-immune serum controls.

### Biopanning protocol and phage extraction

Four rounds of biopanning were done to generate immuno-reactive biopanned libraries. In the first step, parent libraries, fed male, 24 and 96 h fed female phage display libraries were immuno-screened with antibodies to 24 and 48 h fed *A. americanum* tick saliva proteins using a biopanning kit according to instructions by the manufacturer (Novagen, Madison, WI, USA). 96-well EIA/RIA plates (Corning, Corning, NY, USA) were used in our biopanning protocol. Prior to antibody application, wells were rinsed with deionized water several times. In the first step, a 100 μL of serum diluted 1:4 or 1:10 in ELISA Coating Buffer (BioLegend, San Diego, CA, USA) were applied per well and left overnight at 4°C. After antibody binding, wells were washed three times with ELISA coating buffer. Subsequently wells were incubated at 4°C overnight with 200 μL of 5% solution of blocking reagent provided with the kit. Following blocking, wells were washed 5 times with deionized water. Following washing wells were incubated with 1 × 10^8^ pfu (plaque forming units) of each parent library in 100 μL of TBST (Tris-Buffered Saline with 0.05% Tween) overnight at 4°C. Subsequently unbound phages were removed by washing (5×) with TBST. To elute bound phages, wells were incubated with 200 μL of T7 phage elution buffer (0.1% SDS solution) at room temperature for 20 min. Eluted phages were amplified by inoculating 50 mL of host cells (fresh culture of BLT5403 strain in LB medium with cabenicillin, OD_600_ = 0.5-0.6) and incubation at 37°C with shaking until lysis of host cells was observed. The lysate was clarified by centrifugation at 8000 g for 10 minutes. The clarified extract was subjected to phage extraction using 12.5 mL of 20% PEG8000/2.5 M sodium chloride. Pelleted phages were re-suspended in 1 mL of STE buffer. Titer of purified phages was determined as derscribed above. The biopanning protocol was repeated four times to generate immuno-reactive biopanned libraries. To generate false positive controls, parent libraries were also biopanned with rabbit pre-immune serum as described above.

### Next generation sequencing

Parent libraries (fed male, 24 and 96 h fed female libraries) and biopanned libraries were subjected to next generation sequencing using Illumina HiSeq2000 system with the following options: paired-end sequencing, read length of 100 bp, and 800,000 reads per sample. Libraries were prepared for sequencing by PCR amplification using AccuPrime *Pfx* DNA Polymerase (Invitrogen – Life Technologies, Carlsbad, CA, USA). 100 μL PCR reaction contained 2 μL of phages, 10 μL of 10× AccuPrime *Pfx* Reaction Mix, 3 μL of each T7Select UP and T7Select DOWN primer (10 μM), 1 μL of AccuPrime *Pfx* DNA Polymerase (2.5 units/μL), and 81 μL of nuclease free water. PCR cycling conditions were initial denaturation of 2 min at 95°C, followed by 35 cycles for 15 s at 95°C, 30s at 50°C, and 3 min at 68°C. Following amplification, PCR products were purified using MicroElute Cycle-Pure Kit (Omega Bio-Tek, Norcross, GA, USA). Elution was performed in 20 μL of Elution Buffer and samples were stored at −20°C until sequencing started.

### *De novo*assembly and sequence analysis

Sequence reads trimmed at the default .05 limit value were *de novo* assembled using CLC Genomics Workbench software version 6.0.2 (CLC Bio-Qiagen, Cambridge, MA, USA), with all other parameters set to the default settings. Contigs that were found in pre-immune serum biopanned libraries were considered non-specific and eliminated from further analysis. To annotate assembled contigs, sequences were batch scanned against tick sequences in GenBank using BlastX homology search. The Blast search reported in this study was done during March 2014, and there is a possibility that findings may change when new sequences are deposited in GenBank. To identify contigs that were present in multiple biopanned libraries, contig lists were compared using the bl2seq pairwise alignment tool at NCBI. Multiple sequence alignments were done using MacVector version 12 (Mac Vector, Inc., Cary, NC, USA).

## Results and discussion

### Biopanning, next generation sequencing and *de novo*assembly

Three parent phage display cDNA expression libraries, 24 h (PL1) and 96 h (PL2) fed female and fed male (PL3) *A. americanum* ticks, were successfully immuno-screened with antibodies (Ab) to 24 and 48 h fed female tick saliva proteins to generate four biopanned libraries (BPs). The 4 BPs include PL1 biopanned with 24 h Ab (BP6) and 48 h Ab (BP10), PL2 biopanned with 48 h Ab (BP13-14), and PL3 biopanned with 48 h Ab (BP15-16) (Table 1). Biopanning PL1-3 with pre-immune control serum obtained from rabbits prior to tick infestation generated negative control BPs (N-BP1-3) (not shown). PCR amplified PLs, BPs, and N-BPs were successfully sequenced using Illumina HiSeq2000 and individually *de novo* assembled using CLC Genomics Workbench. As summarized in Table 1, biopanning of PL1 (5889 contigs) with Ab to 24 and 48 h *A. americanum* tick saliva proteins yielded 109 (BP6) and 117 (BP10) contigs, respectively. Likewise biopanning of PL2 (6240 contigs) and PL3 (5464 contigs) with antibodies to 48 h *A. americanum* tick saliva proteins yielded 419 (BP13-14) and 266 (BP15-16) contigs, respectively. BlastX analysis showed that all contigs in N-BPs and 16 contigs in biopanned libraries coded for phage proteins. This analysis identified 895 contigs that code putative immunogenic *A. americanum* tick saliva proteins secreted during the first 24-48 h of feeding. The strategy to immuno-screen with antibodies to 24 h *A. americanum* tick saliva proteins was to attempt finding proteins that are injected into the host within 24 h of tick feeding. Additionally immuno-screening male tick phage library identified immunogenic proteins that are shared between female and male ticks. Data in Table 2 show that 56% (60/109) of BP6 contigs were not found in other biopanned libraries, while ~20% (52/266) of BP15-16 contigs were also found in female tick biopanned libraries.

**Table 1:** **Sequence reads and contig numbers in parent (PL) and biopanned (BP) libraries**

Library name	Description	Number of sequence reads	Number of ***de novo***assembled contigs
PL1	24 h fed female tick	1.400.000	5889
PL2	96 h fed female tick	2.000.000	6240
PL3	Fed male tick	1.700.000	5464
BP6	PL1 biopanned with antibodies to 24 h *A. americanum* TSP^1^	3.800.000	109
BP10	PL1 biopanned with antibodies to 48 h *A. americanum* TSP^1^	1.700.000	117
BP13-14	PL2 biopanned with antibodies to 48 h *A. americanum* TSP^1^	4.000.000	419
BP15-16	PL3 biopanned with antibodies to 48 h *A. americanum* TSP^1^	4.200.000	266

^1^Tick saliva proteins.

**Table 2:** ***Amblyomma americanum*** **tick saliva proteins encoding cDNAs conserved in other tick species, but not in mammals**

Accession#	Source library	Top matches in GenBank [accession#]	e-values
GBAI01000007	BP6	unknown larval protein mRNA, complete cds *Rhipicephalus annulatus* [EF675686.1]	1^e-124^
		immunogenic protein mRNA, complete cds *Haemaphysalis longicornis* [GQ499841.1]	5^e-111^
		secreted protein, putative, mRNA *Ixodes scapularis* [XP_002399589.1]	6^e-77^
		Hq05 mRNA, complete cds *Haemaphysalis qinghaiensis* [AY626791.1]	4^e-70^
GBAJ01000081	BP10	unknown larval protein mRNA, complete cds *Rhipicephalus annulatus* [EF675686.1]	1^e-06^
		immunogenic protein mRNA, complete cds *Haemaphysalis longicornis* [GQ499841.1]	0.003
GBAJ01000082	BP10	unknown larval protein mRNA, complete cds *Rhipicephalus annulatus* [EF675686.1]	2^e-09^
		immunogenic protein mRNA, complete cds *Haemaphysalis longicornis* [GQ499841.1]	7^e-07^
GBAI01000013	BP6	mucin-like protein *Dermacentor variabilis* [ACF35532.1]	4^e-40^
		salivary mucin *Amblyomma variegatum* [DAA34695.1]	2^e-24^
		chitinase, putative *Ixodes scapularis* [XP_002404149.1]	6^e-22^
GBAK01000244	BP13-14	mucin-like protein *Dermacentor variabilis* [ACF35532.1]	3^e-41^
		salivary mucin *Amblyomma variegatum* [DAA34695.1]	5^e-24^
		chitinase, putative *Ixodes scapularis* [XP_002404149.1]	4^e-23^
GBAK01000411	BP13-14	Cht mRNA for chitinase, complete cds *Haemaphysalis longicornis* [AB074977.1]	7^e-12^
GBAI01000021	BP6	AV422 mRNA, complete cds *Amblyomma americanum* [KC222016.1]	2^e-08^
GBAK01000213	BP13-14		6
GBAL01000042	BP15-16		7^e-35^
GBAI01000030	BP6	hypothetical secreted protein 1447 mRNA, complete cds *Amblyomma variegatum* [BK007660.1]	4^e-09^
GBAI01000032	BP6	unknown *Haemaphysalis qinghaiensis* [ABQ96857.1]	2^e-11^
GBAJ01000049	BP10		1^e-11^
GBAI01000033	BP6	conserved hypothetical protein *Ixodes scapularis* [XP_002404412.1]	3^e-07^
GBAI01000037	BP6	conserved protein 364 *Amblyomma variegatum* [DAA34231.1]	2^e-12^
GBAI01000051	BP6	hypothetical secreted protein 1652 *Amblyomma variegatum* [DAA34045.1]	2^e-45^
		putative salivary secreted protein *Ixodes scapularis* [AAY66581.1]	4^e-45^
		salivary protein antigen P23 *Ixodes scapularis* (AEE89467.1, 2^e-38^)	1^e-41^
		secreted salivary gland peptide *Ixodes scapularis* [XP_002435217.1]	2^e-38^
GBAI01000092	BP6	conserved hypothetical protein, mRNA *Ixodes scapularis* [XM_002413966.1]^1^	5^e-40^
GBAI01000096	BP6	conserved hypothetical protein *Ixodes scapularis* [XP_002414011.1]	1^e-80^
GBAI01000102	BP6	hypothetical protein *Haemaphysalis longicornis* [BAE02551.1]	7^e-07^
GBAK01000356	BP13-14		3^e-05^
GBAI01000061	BP6	putative cement protein *Amblyomma variegatum* [BK007766.1]	4^e-04^
		conserved hypothetical protein, mRNA *Ixodes scapularis* [XM_002400050.1]	0.3
GBAJ01000018	BP10		0.36
GBAK01000332	BP13-14		0.37
GBAL01000162	BP15-16		0.091
GBAJ01000021	BP10	conserved hypothetical protein *Ixodes scapularis* [XP_002403158.1]	2^e-56^
GBAJ01000051	BP10	hypothetical protein IscW_ISCW013255 *Ixodes scapularis* [XM_002413442.1]	6^e-91^
GBAK01000272	BP13-14	putative cement protein *Amblyomma variegatum* [DAA34732.1]	4^e-12^
GBAJ01000077	BP10		3^e-04^
GBAJ01000078	BP10	hypothetical protein *Haemaphysalis longicornis* [BAE02552.1]	3^e-10^
GBAJ01000079	BP10		2^e-08^
GBAK01000069	BP13-14		4^e-08^
GBAJ01000086	BP10	conserved hypothetical protein *Ixodes scapularis* [XP_002399913.1]	3^e-41^
GBAK01000083	BP13-14	secreted salivary gland peptide, putative *Ixodes scapularis* [XM_002412128.1]	2^e-18^
GBAJ01000091	BP10		6^e-22^
GBAJ01000099	BP10	hypothetical secreted protein 123 mRNA, complete cds *Amblyomma americanum* [BK007643.1]	1^e-10^
GBAK01000035	BP13-14	hypothetical protein IscW_ISCW001430 *Ixodes scapularis* [XP_002401466.1]	1^e-04^
GBAK01000049	BP13-14	mucin peritrophin salivary protein *Amblyomma variegatum* [DAA34644.1]	3^e-05^
GBAK01000045	BP13-14	hypothetical protein IscW_ISCW000843 *Ixodes scapularis* [XP_00240030.1]	3^e-39^
GBAL01000114	BP15-16		1^e-48^
GBAK01000058	BP13-14	*Ixodes scapularis* hypothetical protein, mRNA *Ixodes scapularis* [XM_002412067.1]	4 ^e-21^
GBAK01000062	BP13-14	conserved hypothetical protein, mRNA *Ixodes scapularis* [XM_002411503.1]	2^e-27^
GBAK01000109	BP13-14	hypothetical protein IscW_ISCW011068 *Ixodes scapularis* [XP_002411440.1]	9^e-06^
GBAK01000119	BP13-14	conserved hypothetical protein *Ixodes scapularis* [XP_002416006.1]	1^e-08^
GBAK01000123	BP13-14	hypothetical protein, mRNA *Ixodes scapularis* [XM_002436116.1]	3^e-07^
GBAK01000138	BP13-14	hypothetical protein *Haemaphysalis longicornis* [BAE02708.1]	8^e-07^
GBAK01000161	BP13-14	hypothetical protein, mRNA *Ixodes scapularis* [XM_002400149.1]	3^e-07^
GBAK01000165	BP13-14	conserved hypothetical protein *Ixodes scapularis* [XP_002435325.1]	1^e-27^
GBAK01000185	BP13-14	hypothetical protein IscW_ISCW024828 *Ixodes scapularis* [XP_002416135.1]	7^e-06^
GBAK01000195	BP13-14	secreted PAPA repeat protein *Amblyomma variegatum* [DAA34610.1]	1^e-04^
		hypothetical protein *Ixodes scapularis* [(XP_002433942.1]	6^e-07^
GBAK01000417	BP13-14	conserved hypothetical protein *Ixodes scapularis* [XP_002434340.1]	1^e-10^
GBAK01000238	BP13-14	conserved hypothetical protein *Ixodes scapularis* [XP_002403178.1]	1^e-118^
GBAK01000260	BP13-14	hypothetical secreted protein 94 *Amblyomma variegatum* [DAA34289.1]	4^e-08^
GBAK01000306	BP13-14	hypothetical protein IscW_ISCW024139 *Ixodes scapularis* [XP_002408992.1]	9^e-44^
GBAL01000156	BP15-16		1^e-37^
GBAK01000322	BP13-14	conserved hypothetical protein *Ixodes scapularis* [XP_002399367.1]	4^e-61^
GBAK01000324	BP13-14	cuticle protein 10.9 *Ixodes ricinus* [P84251.1]	1^e-39^
		secreted salivary gland peptide *Ixoders scapularis* [XP_002407787.1]	2^e-39^
GBAK01000338	BP13-14	conserved hypothetical protein *Ixodes scapularis* [XM_002412107.1]	1^e-77^
GBAK01000367	BP13-14	hypothetical protein IscW_ISCW001471 *Ixodes scapularis* [XM_002399258.1]	1^e-26^
GBAL01000163	BP15-16		3^e-41^
GBAK01000374	BP13-14	hypothetical protein IscW_ISCW002509 *Ixodes scapularis* [XP_002403210.1]	5^e-15^
GBAK01000408	BP13-14	CDC73 protein, putative *Ixodes scapularis* [XP_002410866.1]	3^e-46^
GBAK01000350	BP13-14	conserved hypothetical protein *Ixodes scapularis* [XP_002400434.1]^2^	4^e-61^
GBAK01000378	BP13-14	putative secreted salivary protein *Ixodes scapularis* [AAY66509.1]	4^e-09^
GBAL01000023	BP15-16	conserved hypothetical protein *Ixodes scapularis* [XM_002411217.1]	6^e-24^
GBAL01000028	BP15-16	conserved hypothetical protein *Ixodes scapularis* [XP_002404724.1]	1^e-27^
GBAL01000035	BP15-16	putative secreted salivary protein *Ixodes scapularis* [AAY6670.1]	6^e-17^
GBAL01000036	BP15-16	putative salivary secreted peptide *Ixodes pacificus* [AAT92118.1]	8^e-35^
		secreted salivary gland peptide, putative *Ixodes scapularis* [XP_002433339.1]	7^e-34^
GBAL01000048	BP15-16	conserved hypothetical protein *Ixodes scapularis* [XM_002407819.1]	1^e-09^
GBAK01000098	BP13-14		1^e-09^
GBAL01000057	BP15-16	conserved hypothetical protein *Ixodes scapularis* [XM_002409429.1]	2^e-09^
GBAL01000078	BP15-16	conserved hypothetical protein *Ixodes scapularis* [XM_002415769.1]	0.008
GBAL01000118	BP15-16	putative cement protein mRNA, complete cds *Amblyomma variegatum* [BK007766.1]	6^e-35^
GBAL01000126	BP15-16	conserved hypothetical protein *Ixodes scapularis* [XP_002406503.1]	3^e-74^
GBAL01000148	BP15-16	conserved hypothetical protein *Ixodes scapularis* [XP_002413922.1]	2^e-76^
GBAL01000168	BP15-16	clone HqL09 unknown mRNA *Haemaphysalis qinghaiensis* [EF605265.1]	9^e-19^
GBAL01000169	BP15-16	hypothetical protein IscW_ISCW007130 *Ixodes scapularis* [XP_002403722.1]	3^e-13^
GBAL01000181	BP15-16	ubiquitously expressed transcript (UXT), putative *Ixodes scapularis* [XP_002410385.1]	5^e-56^
GBAL01000185	BP15-16	conserved hypothetical protein *Ixodes scapularis* [XP_002400069.1]	6^e-05^
GBAL01000196	BP15-16	hypothetical protein IscW_ISCW011816 *Ixodes scapularis* [XP_002412243.1]	2^e-10^
GBAL01000210	BP15-16	secreted protein, putative *Ixodes scapularis* [XP_002401383.1]	1^e-27^
GBAL01000221	BP15-16	secreted salivary gland peptide, putative *Ixodes scapularis* [XP_002401305.1]	1^e-22^
GBAL01000247	BP15-16	hypothetical protein IscW_ISCW011424 *Ixodes scapularis* [XP_002412199.1]	3^e-16^
GBAL01000249	BP15-16	secreted salivary gland peptide, putative *Ixodes scapularis* [XP_002414543.1]	4^e-06^
GBAL01000252	BP15-16	secreted protein, putative *Ixodes scapularis* [XP_002408033.1]	0.29
		glycine rich protein 44 *Amblyomma variegatum* [DAA34246.1]	0.98
GBAL01000253	BP15-16	65-kDa macrophage protein, putative *Ixodes scapularis* [XP_002413054.1]	2^e-42^
GBAL01000255	BP15-16	conserved hypothetical protein *Ixodes scapularis* [XM_002433956.1]	3^e-42^
GBAL01000260	BP15-16	conserved hypothetical protein *Ixodes scapularis* [XM_002435514.1]	1^e-36^

^1^show conservation in mammals.

^2^highly identical to apoptosis response protein.

Of the 895 contigs from the *A. americanum* immuno-transcriptome, 431 (listed in an Additional file 1) did not show amino acid identities to previously annotated proteins in GenBank, while the remaining 464 sequences were provisionally annotated on the basis of their identity to protein sequences in GenBank. The provisionally annotated sequences include cross-tick species conserved orphan tick saliva proteins (Table 2), proteases (Table 3), protease inhibitors (Table 4), transporters and/or binding proteins (Table 5), enzymes (Table 6), ribosomal proteins (Table 7), and proteins of miscellaneous functions (Table 8). For clarity, the rest of this discussion is arranged under the different classes of provisionally identified proteins in this study.

**Table 3:** **Putative proteases in** ***Amblyomma americanum*** **tick saliva**

Accession#	Source library	Top matches in GenBank [accession#]	e-values
GBAK01000372	BP13-14	putative legumain-like protease precursor *Dermacentor variabilis* [ACF35522.1]	3^e-47^
GBAI01000003	BP6	protease, putative *Ixodes scapularis* [XP_002413749.1]	4^e-40^
		leukotriene hydrolase *Argas monolakensis* [ABI52802.1]	4^e-32^
GBAJ01000102	BP10	protease, putative *Ixodes scapularis* [XP_002413749.1]	2^e-26^
		leukotriene hydrolase *Argas monolakensis* [ABI52802.1]	3^e-21^
GBAK01000094	BP13-14	putative cathepsin B-like cysteine protease form 1 *Dermacentor variabilis* [ACF35525.1]	5^e-60^
		cathepsin B-like cysteine protease form 1 *Ixodes ricinus* [ABO26563.1]	3^e-56^
GBAI01000024	BP6	cathepsin L-like cysteine proteinase A *Rhipicephalus haemaphysaloides haemaphysaloides* [AAQ16117.1]	3^e-14^
GBAJ01000008	BP10		2^e-82^
GBAK01000180	BP13-14		3^e-28^
GBAK01000182	BP13-14	Longipain *Haemaphysalis longicornis* [BAF43801.1]	4^e-55^
GBAK01000183	BP13-14		7^e-41^
GBAK01000214	BP13-14	conserved hypothetical protein *Ixodes scapularis* [XP_002414190.1]	7^e-15^
		serine carboxypeptidase, putative *Ixodes scapularis* [XP_002403464.1]	2^e-12^
GBAL01000134	BP15-16	conserved hypothetical protein *Ixodes scapularis* [XP_002414190.1]	2^e-13^
		serine carboxypeptidase, putative *Ixodes scapularis* [XP_002414193.1]	8^e-11^
GBAJ01000040	BP10	serine carboxypeptidase, putative *Ixodes scapularis* [XP_002403464.1]	2^e-14^
GBAK01000358	BP13-14	angiotensin-converting enzyme, putative *Ixodes scapularis* [XP_002401260.1]	2^e-40^
GBAK01000026	BP13-14	neprilysin, putative *Ixodes scapularis* [XP_002404392.1]	1^e-42^
GBAK01000196	BP13-14	neprilysin, putative *Ixodes scapularis* [XP_002414107.1]	1^e-05^
GBAK01000111	BP13-14	metallopeptidase *Amblyomma variegatum* [DAA34047.1]	4^e-81^
GBAK01000132	BP13-14	metalloprotease *Haemaphysalis longicornis* [BAE72664.1]	3^e-06^
		metalloprotease, putative *Ixodes scapularis* [XP_002407430.1]	4^e-06^
		metalloprotease *Argas monolakensis* [ABI52779.1]	3^e-04^
GBAK01000269	BP13-14	ubiquitin fusion-degradation protein, putative *Ixodes scapularis* [XP_002414671.1]	5^e-62^

**Table 4:** **Putative protease inhibitors in** ***Amblyomma americanum*** **tick saliva**

Accession#	Source library	Top matches in GenBank [accession#]	e-values
GBAI01000043	BP6	neutrophil elastase inhibitor *Rhipicephalus microplus* [ABH10604.1]	2^e-14^
GBAI01000058	BP6		3^e-07^
GBAJ01000027	BP10		2^e-14^
GBAL01000059	BP15-16		9^e-11^
GBAK01000097	BP13-14	hypothetical protein *Haemaphysalis longicornis* [BAE02553.1]	2^e-12^
GBAL01000201	BP15-16		3^e-12^
GBAK01000040	BP13-14	Chymotrypsin-elastase inhibitor ixodidin *Rhipicephalus microplus* [P83516]	2^e-06^
GBAJ01000043	BP10	Kunitz-like protease inhibitor precursor *Amblyomma variegatum* [DAA34606.1]	2^e-29^
GBAK01000277	BP13-14	putative salivary protein with Kunitz domains *Ixodes scapularis* [AAY66736.1]	4^e-04^
GBAJ01000023	BP10	putative Kunitz-BPTI protein *Dermacentor variabilis* [ACF35511.1]	1^e-13^
GBAK01000091	BP13-14		2^e-13^
GBAK01000073	BP13-14	Carboxypeptidase inhibitor *Rhipicephalus bursa* [Q5EPH2.1]	3^e-33^
GBAJ01000071	BP10	ATPase inhibitor, putative *Ixodes scapularis* [XP_002399280.1]	5^e-29^
GBAK01000345	BP13-14		9^e-26^
GBAK01000027	BP13-14	translation initiation inhibitor UK114/IBM1, putative *Ixodes scapularis* [XP_002434004.1]	2^e-12^
GBAL01000098	BP15-16		2^e-12^
GBAK01000064	BP13-14	cystatin *Haemaphysalis longicornis* [ABZ89554.1]	2^e-63^
		cystatin *Dermacentor silvarum* [ADZ23478.1]	4^e-62^
		cystatin 2c *Rhipicephalus microplus* [AGW80659.1]	1^e-59^
GBAL01000180	BP15-16	cystatin 2b *Rhipicephalus microplus* [AGW80658.1]	3^e-51^
		gut cystatin *Rhipicephalus appendiculatus* [AGB35873.1]	3^e-51^
		putative secreted cystatin *Dermacentor variabilis* [ACF35514.1]	3^e-36^
GBAL01000013	BP15-16	cystatin *Haemaphysalis longicornis* [ABV71390.1]	8^e-14^

**Table 5:** **Putative immunogenic binding proteins and transporters present in** ***Amblyomma americanum*** **tick saliva**

Accession#	Source library	Top matches in GenBank [accession#]	e-values
GBAJ01000035	BP10	hemelipoglycoprotein precursor, mRNA, complete cds *Dermacentor variabilis* [DQ422963.1]	0.085
GBAL01000006	BP15-16	heme lipoprotein precursor, mRNA, complete cds *Amblyomma americanum* [EF050790.3]	0.43
GBAK01000315	BP13-14		4^e-50^
GBAI01000078	BP6	ferritin *Amblyomma americanum* [AAQ54708.1]	7^e-79^
GBAJ01000036	BP10		3^e-61^
GBAJ01000098	BP10		2^e-45^
GBAK01000211	BP13-14		3^e-96^
GBAL01000165	BP15-16		1^e-37^
GBAJ01000019	BP10	ferritin *Haemaphysalis longicornis* [AAQ54713.1]	2^e-17^
GBAI01000083	BP6	calmodulin, putative *Ixodes scapularis* [XP_002404770.1]	3^e-53^
GBAJ01000110	BP10		7^e-68^
GBAK01000031	BP13-14		7^e-78^
GBAL01000053	BP15-16		1^e-77^
GBAJ01000101	BP10	calreticulin (crt-1) mRNA, complete cds *Amblyomma americanum* [U07708.1]	3^e-10^
GBAK01000025	BP13-14		7^e-25^
GBAI01000101	BP6	calponin, putative *Ixodes scapularis* [XP_002402437.1]	1^e-56^
GBAL01000044	BP15-16	sarcoplasmic calcium-binding proteins I, III, and IV, putative *Ixodes scapularis* [XP_002434211.1]	1^e-04^
GBAK01000066	BP13-14	lipocalin *Argas monolakensis* [ABI152816.1]	0.002
GBAK01000093	BP13-14	salivary lipocalin *Amblyomma variegatum* [DAA34666.1]	3^e-06^
GBAL01000248	BP15-16		1^e-07^
GBAI01000035	BP6	salivary lipocalin *Amblyomma variegatum* [DAA34698.1]	9^e-30^
GBAK01000144	BP13-14		2^e-05^
GBAL01000122	BP15-16		4^e-04^
GBAK01000151	BP13-14	serotonin and histamine binding protein *Dermacentor reticulatus* [AAL56644.1]	7^e-19^
GBAL01000133	BP15-16	lipocalin *Argas monolakensis* [ABI52807.1]	6^e-10^
GBAJ01000056	BP10	fatty acid-binding protein FABP *Amblyomma variegatum* [DAA34565.1]	3^e-06^
GBAL01000186	BP15-16	ATP binding protein, putative *Ixodes scapularis* [XP_002399785.1]	0.49
GBAK01000068	BP13-14	GTP-binding protein, putative *Ixodes scapularis* [XP_002412036.1]	1^e-07^
GBAJ01000103	BP10	histidine triad (hit) protein, putative *Ixodes scapularis* [XP_002412911.1]	6^e-48^
GBAL01000222	BP15-16	RNA-binding protein, putative *Ixodes scapularis* [XP_002410612.1]^1^	2.1
GBAL01000072	BP15-16	RNA-binding nuclear protein, putative *Ixodes scapularis* [XP_002413820.1]	6^e-10^
GBAL01000055	BP15-16	RNA recognition motif protein, putative *Ixodes scapularis* [XP_002411413.1]	2^e-36^
GBAK01000395	BP13-14	DNA-binding protein C1D *Amblyomma variegatum* [DAA34443.1]	2^e-31^
		sun-cor steroid hormone receptor co-repressor, putative *Ixodes scapularis* [XP_002402463.1]	4^e-26^
GBAK01000082	BP13-14	immunoglobulin G binding protein A *Rhipicephalus appendiculatus* [AAB68801.1]	3^e-58^
GBAK01000159	BP13-14		9^e-10^
GBAK01000246	BP13-14	insulin-like growth factor binding protein-related protein 6 long mRNA, complete cds *Amblyomma americanum* [GU907779.1]	1^e-49^
		insulin-like growth factor binding protein-related protein 6 short mRNA, complete cds *Amblyomma americanum* [GU907778.1]	5^e-46^
GBAL01000113	BP15-16	Evasin-1 *Rhipicephalus sanguineus* [E0C8P7.1]	6^e-08^
GBAL01000188	BP15-16	actin-binding protein Sla2, putative *Ixodes scapularis* [XP_002434768.1]	2^e-74^
GBAK01000236	BP13-14	cyclophilin A *Haemaphysalis longicornis* [BAG41813.1]	4^e-81^
GBAL01000024	BP15-16		4^e-81^
GBAK01000404	BP13-14	monocarboxylate transporter, putative *Ixodes scapularis* [XP_002435530.1]	0.003
GBAL01000241	BP15-16	phosphatidylcholine transfer protein, putative *Ixodes scapularis* [XP_002408227.1]	4^e-43^
GBAK01000116	BP13-14	translocon-associated complex TRAP, alpha subunit, putative *Ixodes scapularis* [XP_002413372.1]	2.2
GBAL01000016	BP15-16	trafficking protein particle complex subunit 6B, putative *Ixodes scapularis* [XP_002402611.1]	8^e-54^
GBAK01000379	BP13-14		9^e-54^

^1^contain G patch domain.

**Table 6:** **Putative immunogenic enzymes in** ***Amblyomma americanum*** **tick saliva**

Accession#	Source library	Top matches in GenBank [accession#]	e-values
GBAI01000009	BP6	FoF1 ATPase subunit6 *Amblyomma americanum* [ABA19091.1]	9^e-57^
GBAJ01000009	BP10	F1F0 ATP-synthase subunit Cf6, putative *Ixodes scapularis* [XP_002399676.1]	8^e-55^
GBAI01000048	BP6		7^e-46^
GBAK01000355	BP13-14	vacuolar H + −ATPase V1 sector, subunit G, putative *Ixodes scapularis* [XP_002415521.1]	1^e-43^
GBAK01000200	BP13-14	ADP/ATP translocase *Ixodes scapularis* [AAY66969.1]	9^e-19^
GBAK01000110	BP13-14	inner mitochondrial membrane translocase TIM17-like protein, partial *Ixodes scapularis* [AAY66838.1]	5^e-40^
GBAK01000320	BP13-14	mitochondrial malate dehydrogenase, partial *Ixodes scapularis* [AAY66975.1]	3^e-34^
GBAI01000028	BP6	C1-tetrahydrofolate synthase, putative *Ixodes scapularis* [XP_002401635.1]	2^e-39^
GBAK01000202	BP13-14	cytochrome oxidase subunit 1 *Amblyomma americanum* [ABA19092.1]	4^e-74^
GBAL01000140	BP15-16		5^e-43^
GBAI01000098	BP6	COX1 gene product (mitochondrion) *Bothriocroton concolor* [YP_006234392.1]	9^e-47^
GBAI01000099	BP6	cytochrome c oxidase subunit I *Rhipicephalus zambeziensis* [AAG23880.1]	2^e-40^
GBAK01000263	BP13-14	cytochrome c oxidase subunit I *Amblyomma limbatum* [ACM17834.1]	1^e-59^
GBAL01000112	BP15-16	cytochrome oxidase subunit 1 *Ixodes scapularis* [ADO64507.1]	6^e-55^
GBAL01000084	BP15-16	cytochrome c oxidase subunit I *Rhipicephalus maculatus* [AAG23878.1]	4^e-25^
GBAL01000143	BP15-16	cytochrome oxidase subunit 2 *Amblyomma americanum* [ABA19093.1]	2^e-42^
GBAK01000067	BP13-14		3^e-70^
GBAK01000121	BP13-14	cytochrome oxidase subunit 3 *Amblyomma americanum* [ABA19094.1]	1^e-68^
GBAL01000031	BP15-16		9^e-69^
GBAK01000343	BP13-14	cytochrome c oxidase polypeptide IV *Ixodes scapularis* [AAY66918.1]	3^e-55^
GBAK01000282	BP13-14	cytochrome c oxidase polypeptide Vb *Ixodes scapularis* [AAY66932.1]	2^e-37^
GBAJ01000050	BP10	cytochrome C oxidase, subunit VIb/COX12, putative *Ixodes scapularis* [XP_002416556.1]	1^e-07^
GBAK01000008	BP13-14	cytochrome oxidase subunit VIIc *Ixodes pacificus* [AAT92215.1]	2^e-32^
GBAJ01000041	BP10	ubiquinol cytochrome c reductase subunit QCR7 *Amblyomma variegatum* [DAA34591.1]	7^e-52^
GBAK01000326	BP13-14		4^e-47^
GBAJ01000024	BP10	NADH:ubiquinone oxidoreductase, NDUFS2/49 kDa subunit, putative *Ixodes scapularis* [XP_002404495.1]	3^e-82^
GBAJ01000104	BP10	NADH-ubiquinone oxidoreductase ashi subunit, putative *Ixodes scapularis* [XP_002409125.1]	2^e-08^
GBAK01000386	BP13-14	estradiol 17-beta-dehydrogenase, putative *Ixodes scapularis* [XP_002434666.1]	5^e-33^
GBAL01000014	BP15-16	NADH dehydrogenase subunit 1 (mitochondrion) *Amblyomma cajennense* [YP_007475022.1]	3^e-40^
GBAK01000139	BP13-14	NADH dehydrogenase subunit 2 *Amblyomma americanum* [ABA19096.1]	3^e-10^
GBAK01000133	BP13-14	NADH dehydrogenase subunit 3 (mitochondrion) *Amblyomma cajennense* [YP_007475021.1]	5^e-27^
GBAL01000089	BP15-16	NADH dehydrogenase subunit 4 *Amblyomma americanum* [ABA19099.1]	3^e-33^
GBAI01000088	BP6	alkyl hydroperoxide reductase, thiol specific antioxidant, putative *Ixodes scapularis* [XP_002405466.1]	2^e-42^
		thioredoxin peroxidase *Ornithodoros parkeri* [ABR23404.1]	1^e-41^
GBAL01000174	BP15-16	peroxidase, putative *Ixodes scapularis* [XP_002404935.1]	1^e-34^
GBAL01000029	BP15-16	thioredoxin reductase, putative *Ixodes scapularis* [XP_002404402.1]	1^e-68^
GBAI01000050	BP6	putative glutathione S-transferase *Dermacentor variabilis* [ACF35539.1]	4^e-94^
GBAK01000166	BP13-14		7^e-105^
GBAL01000121	BP15-16		7^e-67^
GBAL01000175	BP15-16		1^e-32^
GBAK01000413	BP13-14	putative glutathione S-transferase *Dermacentor variabilis* [ACF35505.1]	9^e-32^
GBAK01000042	BP13-14	gamma-glutamyltransferase, putative *Ixodes scapularis* [XP_002407102.1]	0.006
GBAL01000137	BP15-16		0.006
GBAL01000157	BP15-16	protein disulfide isomerase *Amblyomma variegatum* [ABD16189.1]	0
GBAK01000105	BP13-14		0
GBAK01000289	BP13-14	protein disulfide isomerase *Amblyomma variegatum* [DAA34067.1]	4^e-57^
GBAI01000054	BP6	protein disulfide isomerase *Haemaphysalis longicornis* [ABS50238.1]	1^e-24^
GBAI01000015	BP6	protein disulfide isomerase-1 *Haemaphysalis longicornis* [BAF63672.1]	9^e-04^
GBAK01000337	BP13-14	protein disulfide isomerase-2 *Haemaphysalis longicornis* [BAF63671.1]	3^e-39^
GBAI01000074	BP6	sulfotransferase, putative *Ixodes scapularis* [XP_002435996.1]	9^e-14^
GBAJ01000030	BP10		3^e-19^
GBAL01000144	BP15-16		0.033
GBAJ01000034	BP10	sulfotransferase, putative *Ixodes scapularis* [XP_002436296.1]	7^e-14^
GBAL01000136	BP15-16	sulfotransferase, putative *Ixodes scapularis* [XP_002400534.1]	3^e-35^
GBAK01000319	BP13-14	glycosyl transferase, putative *Ixodes scapularis* [XP_002434372.1]	2^e-31^
GBAK01000388	BP13-14		1^e-38^
GBAK01000266	BP13-14	acyl-CoA synthetase, putative *Ixodes scapularis* [XP_002401840.1]	2^e-31^
GBAK01000079	BP13-14	adenylosuccinate lyase, putative *Ixodes scapularis* [XP_002399354.1]	4^e-34^
GBAK01000191	BP13-14	casein kinase, putative *Ixodes scapularis* [XP_002400161.1]	2^e-30^
GBAL01000108	BP15-16		1^e-30^
GBAK01000072	BP13-14	3-hydroxyacyl-CoA dehydrogenase, putative *Ixodes scapularis* [XP_002415080.1]	1^e-79^
GBAI01000093	BP6	malonyl CoA-acyl carrier protein transacylase, putative *Ixodes scapularis* [XP_002402213.1]	5^e-15^
GBAI01000059	BP6	dihydrolipoamide acetyltransferase, putative *Ixodes scapularis* [XP_002401656.1]	8^e-05^
GBAJ01000090	BP10	RAB GTPase-activating protein, putative *Ixodes scapularis* [XP_002407414.1]	2^e-29^
		GTPase-activating protein *Amblyomma variegatum* [DAA34545.1]	3^e-27^
GBAK01000234	BP13-14	ubiquitin protein ligase *Ixodes scapularis* [XP_002434331.1]	2^e-28^
GBAI01000014	BP6	SCF ubiquitin ligase Skp1 component *Amblyomma variegatum* [DAA34559.1]	2^e-46^
		SCF ubiquitin ligase complex *Ixodes scapularis* [AAY66893.1]	2^e-46^
GBAK01000329	BP13-14	phosphoribosylformylglycinamidine synthase, putative *Ixodes scapularis* [XP_002405859.1]	2^e-35^
GBAK01000254	BP13-14	lysine-ketoglutarate reductase/saccharopine dehydrogenase *Haemaphysalis longicornis* [BAI44335.1]	6^e-77^
		lysine-ketoglutarate reductase, putative *Ixodes scapularis* [XP_002404033.1]	3^e-70^
GBAK01000268	BP13-14	pterin-4-alpha-carbinolamine dehydratase, putative *Ixodes scapularis* [XP_002399841.1]	1^e-56^
GBAK01000157	BP13-14	ornithine aminotransferase, putative *Ixodes scapularis* [XP_002406120.1]	2^e-45^
GBAI01000082	BP6	methionyl-tRNA synthetase, putative *Ixodes scapularis* [XP_002433760.1]	6^e-32^
GBAK01000365	BP13-14	aspartyl-tRNA synthetase, putative *Ixodes scapularis* [XP_002404413.1]	6^e-39^
GBAL01000251	BP15-16	cysteine synthase, putative *Ixodes scapularis* [XP_002415219.1]	3^e-39^
GBAL01000103	BP15-16	ng,ng-dimethylarginine dimethylaminohydrolase, putative, mRNA *Ixodes scapularis* [XM_002404864.1]	1^e-05^
GBAL01000038	BP15-16	keratinocyte transglutaminase, putative *Ixodes scapularis* [XP_002402412.1]	3^e-46^
GBAK01000354	BP13-14		2^e-36^
GBAK01000325	BP13-14	lysosomal acid phosphatase *Haemaphysalis longicornis* [ADN34299.1]	9^e-66^
GBAJ01000044	BP10	triosephosphate isomerase *Rhipicephalus microplus* [AFP81689.1]	8^e-103^
GBAL01000080	BP15-16	phosphoenolpyruvate carboxykinase *Rhipicephalus microplus* [ABO61883.1]	2^e-36^
		phosphoenolpyruvate carboxykinase, putative *Ixodes scapularis* [XP_002404833.1]	1^e-32^
GBAK01000024	BP13-14	D-dopachrome tautomerase, putative *Ixodes scapularis* [XP_002401879.1]	2^e-10^
GBAJ01000109	BP10	transposase, putative *Ixodes scapularis* [XP_002415790.1]	8^e-19^
GBAI01000002	BP6	ribosomal protein S6 kinase, putative *Ixodes scapularis* [XP_002405308.1]	3^e-21^

**Table 7:** **Ribosomal proteins in** ***Amblyomma americanum*** **tick saliva**

Accession#	Source library	Top matches in GenBank [accession#]	e-values
GBAI01000031	BP6	ribosomal protein *Haemaphysalis qinghaiensis* [ACD50888.1]	2^e-37^
		ribosomal protein, putative *Ixodes scapularis* [XP_002400902.1]	1^e-34^
		ribosomal protein, large P2 *Ixodes pacificus* [AAT92169.1]	3^e-34^
GBAK01000179	BP13-14	ribosomal protein *Haemaphysalis qinghaiensis* [ACD50888.1]	2^e-39^
		ribosomal protein, putative *Ixodes scapularis* [XP_002400902.1]	3^e-34^
		ribosomal protein, large P2 *Ixodes pacificus* [AAT92169.1]	7^e-34^
GBAI01000107	BP6	60S ribosomal protein L2/L8 *Ornithodoros coriaceus* [ACB70396.1]	2^e-63^
GBAK01000392	BP13-14		1^e-52^
GBAI01000079	BP6	ribosomal protein L3, putative *Ixodes scapularis* [XP_002416193.1]	6^e-73^
GBAJ01000010	BP10		2^e-72^
GBAK01000172	BP13-14		5^e-87^
GBAL01000021	BP15-16		2^e-88^
GBAL01000022	BP15-16		1^e-36^
GBAL01000146	BP15-16		2^e-10^
GBAI01000049	BP6	60S ribosomal protein L5, putative *Ixodes scapularis* [XP_002434050.1]	1^e-23^
GBAJ01000073	BP10	60S ribosomal protein L9, putative *Ixodes scapularis* [XP_002407167.1]	4^e-118^
GBAK01000059	BP13-14		3^e-48^
GBAK01000198	BP13-14	ribosomal protein L9, putative *Ixodes scapularis* [XP_002433785.1]	2^e-10^
GBAJ01000084	BP10	60S ribosomal protein L10a *Ixodes scapularis* [AAY66960.1]	6^e-41^
GBAK01000385	BP13-14		1^e-54^
GBAL01000009	BP15-16	60S ribosomal protein L10A, putative *Ixodes scapularis* [XP_002404773.1]	3^e-135^
GBAJ01000105	BP10	60S ribosomal protein L10, putative *Ixodes scapularis* [XP_002399224.1]	2^e-56^
GBAI01000057	BP6	ribosomal protein L11, putative *Ixodes scapularis* [XP_002409414.1]	2^e-92^
GBAK01000020	BP13-14		3^e-125^
GBAJ01000028	BP10	ribosomal protein L12 *Dermacentor variabilis* [ACF35542.1]	1^e-102^
GBAK01000299	BP13-14	putative 60S ribosomal protein L13a *Amblyomma maculatum* [ADC97464.1]	2^e-129^
GBAJ01000052	BP10	putative 60S ribosomal protein L13e *Amblyomma americanum* [ADC97461.1]	2^e-97^
GBAK01000188	BP13-14	60S ribosomal protein L14, putative *Ixodes scapularis* [XP_002403086.1]	4^e-19^
GBAL01000051	BP15-16	ribosomal protein L15, putative *Ixodes scapularis* [XP_002402071.1]	8^e-51^
GBAI01000047	BP6	ribosomal protein L17, putative *Ixodes scapularis* [XP_002435561.1]	7^e-100^
GBAJ01000080	BP10	60S ribosomal protein L17 *Ixodes scapularis* [Q4PM54.1]	2^e-30^
GBAK01000155	BP13-14		7^e-48^
GBAI01000036	BP6	ribosomal protein L18a *Ixodes scapularis* [AAY66898.1]	1^e-115^
GBAK01000375	BP13-14		1^e-32^
GBAI01000086	BP6	ribosomal protein L19 *Ixodes scapularis* [AAY66930.1]	3^e-10^
GBAK01000273	BP13-14		3^e-10^
GBAI01000052	BP6	ribosomal protein L21, putative *Ixodes scapularis* [XP_002403588.1]	3^e-87^
GBAJ01000002	BP10		1^e-104^
GBAK01000009	BP13-14		8^e-105^
GBAL01000033	BP15-16		3^e-44^
GBAI01000065	BP6	ribosomal protein L22, putative *Ixodes scapularis* [XP_002412444.1]	5^e-47^
GBAJ01000088	BP10	ribosomal protein L23 *Haemaphysalis qinghaiensis* [AAY42210.1]	7^e-69^
GBAK01000232	BP13-14		3^e-29^
GBAL01000079	BP15-16	60S ribosomal protein L23 *Ixodes scapularis* [AAY66949.1]	3^e-45^
GBAK01000114	BP13-14	60S ribosomal protein L24, putative *Ixodes scapularis* [XP_002409587]	2^e-32^
GBAI01000006	BP6	ribosomal protein L26 *Ixodes scapularis* [AAY66956.1]	6^e-62^
GBAI01000053	BP6	ribosomal protein L27A, putative *Ixodes scapularis* [XP_002411588.1]	3^e-72^
GBAJ01000076	BP10		8^e-74^
GBAJ01000039	BP10	60S ribosomal protein L27, putative *Ixodes scapularis* [XP_002434022.1]	2^e-81^
GBAK01000327	BP13-14		1^e-57^
GBAI01000089	BP6	ribosomal protein L28, putative *Ixodes scapularis* [XP_002406865.1]	2^e-40^
GBAJ01000094	BP10		3^e-39^
GBAK01000004	BP13-14		4^e-19^
GBAL01000085	BP15-16		6^e-40^
GBAI01000022	BP6	60S ribosomal protein L29 *Ornithodoros parkeri* [ABR23378.1]	9^e-27^
GBAJ01000054	BP10		5^e-27^
GBAK01000235	BP13-14		4^e-27^
GBAL01000073	BP15-16		9^e-27^
GBAI01000080	BP6	ribosomal protein L30 *Ixodes pacificus* [AAT92174.1]	2^e-77^
GBAK01000061	BP13-14		6^e-72^
GBAI01000026	BP6	ribosomal protein L31, putative *Ixodes scapularis* [XP_002403582.1]	5^e-26^
		ribosomal protein L31 *Dermacentor variabilis* [ACF35537.1]	6^e-25^
		ribosomal protein L31 *Argas monolakensis* [ABI52770.1]	1^e-23^
GBAK01000248	BP13-14	ribosomal protein L31, putative *Ixodes scapularis* [XP_002403582.1]	9^e-15^
		ribosomal protein L31 *Dermacentor variabilis* [ACF35537.1]	2^e-15^
		ribosomal protein L31 *Argas monolakensis* [ABI52770.1]	3^e-15^
GBAK01000249	BP13-14	ribosomal protein L31, putative *Ixodes scapularis* [XP_002403582.1]	2^e-54^
		ribosomal protein L31 *Dermacentor variabilis* [ACF35537.1]	1^e-57^
		ribosomal protein L31 *Argas monolakensis* [ABI52770.1]	2^e-54^
GBAL01000017	BP15-16	ribosomal protein L31, putative *Ixodes scapularis* [XP_002403582.1]	1^e-68^
		ribosomal protein L31 *Dermacentor variabilis* [ACF35537.1]	5^e-72^
		ribosomal protein L31 *Argas monolakensis* [ABI52770.1]	1^e-68^
GBAI01000075	BP6	60S ribosomal protein L32, putative *Ixodes scapularis* [XP_002399507.1]	2^e-47^
GBAK01000076	BP13-14		9^e-57^
GBAL01000139	BP15-16		4^e-56^
GBAL01000177	BP15-16		7^e-25^
GBAI01000067	BP6	ribosomal protein L34 *Dermacentor variabilis* [ACF35536.1]	9^e-60^
GBAJ01000005	BP10		1^e-78^
GBAJ01000007	BP10		7^e-74^
GBAL01000158	BP15-16		4^e-59^
GBAK01000216	BP13-14	60s ribosomal protein L34 *Ornithodoros parkeri* [ABR23475.1]	0.079
GBAI01000084	BP6	60S ribosomal protein L35-like protein *Dermacentor variabilis* [ACF35541.1]	1^e-50^
GBAJ01000065	BP10		1^e-56^
GBAL01000099	BP15-16		4^e-62^
GBAI01000104	BP6	ribosomal protein L35a *Ixodes scapularis* [AAY66948.1]	4^e-54^
GBAK01000296	BP13-14		4^e-66^
GBAK01000203	BP13-14	ribosomal protein L37A *Ornithodoros parkeri* [ABR23427.1]	6^e-58^
		ribosomal protein L37A *Ixodes scapularis* [AAY66836.1]	6^e-58^
GBAI01000108	BP6	60S ribosomal protein L37 *Ixodes scapularis* [AAY66940.1]	5^e-41^
GBAJ01000022	BP10		2^e-43^
GBAK01000262	BP13-14		7^e-20^
GBAK01000291	BP13-14	ribosomal protein L39 *Ixodes scapularis* [AAY66991.1]	8^e-30^
GBAK01000220	BP13-14	ribosomal protein L40, putative *Ixodes scapularis* [XP_002401964.1]	6^e-60^
GBAI01000044	BP6	60S ribosomal protein L44 *Ornithodoros parkeri* [ABR23416.1]	1^e-54^
GBAJ01000037	BP10		1^e-47^
GBAK01000071	BP13-14		1^e-69^
GBAI01000068	BP6	40S ribosomal protein S2/30S *Ornithodoros parkeri* [ABR23354.1]	2^e-46^
		40S ribosomal protein, putative *Ixodes scapularis* [XP_002400781.1]	6^e-46^
GBAL01000191	BP15-16	40S ribosomal protein S2/30S *Ornithodoros parkeri* [ABR23354.1]	2^e-58^
		40S ribosomal protein, putative *Ixodes scapularis* [XP_002400781.1]	4^e-59^
GBAI01000001	BP6	40S ribosomal protein S3 *Ornithodoros parkeri* [ABR23477.1]	4^e-36^
GBAJ01000042	BP10		2^e-36^
GBAK01000305	BP13-14		2^e-53^
GBAK01000100	BP13-14	40S ribosomal protein S3a *Amblyomma variegatum* [DAA34106.1]	3^e-92^
GBAK01000101	BP13-14	40S ribosomal protein S3a *Argas monolakensis* [ABI52667.1]	8^e-28^
GBAL01000166	BP15-16		5^e-35^
GBAK01000303	BP13-14	ribosomal protein S4 *Ornithodoros parkeri* [ABR23501.1]	2^e-50^
GBAL01000235	BP15-16	40S ribosomal protein S4 *Ixodes scapularis* [Q4PMB3.1]	6^e-78^
GBAK01000108	BP13-14	40S ribosomal protein S5 *Dermacentor variabilis* [AAO92286.1]	2^e-143^
GBAK01000383	BP13-14	truncated 40S ribosomal protein S7 *Amblyomma variegatum* [DAA34567.1]	4^e-43^
		40S ribosomal protein S7, putative *Ixodes scapularis* [XP_002405269.1]	9^e-41^
GBAK01000050	BP13-14	ribosomal protein S8, putative *Ixodes scapularis* [XP_002400259.1]	1^e-106^
GBAJ01000003	BP10	40S ribosomal protein S12 *Dermacentor variabilis* [AAP04352.1]	2^e-91^
GBAK01000242	BP13-14		7^e-80^
GBAL01000145	BP15-16		1^e-91^
GBAJ01000074	BP10	40S ribosomal protein S14, putative *Ixodes scapularis* [XP_002415092.1]	5^e-57^
GBAK01000028	BP13-14		2^e-82^
GBAI01000064	BP6	ribosomal protein S15Aa *Ixodes scapularis* [AAY66923.1]	3^e-65^
GBAJ01000026	BP10		3^e-63^
GBAI01000097	BP6	40S ribosomal protein S15, putative *Ixodes scapularis* [XP_002406430.1]	8^e-41^
GBAJ01000100	BP10		1^e-54^
GBAK01000141	BP13-14		2^e-54^
GBAL01000182	BP15-16		4^e-28^
GBAI01000062	BP6	acidic ribosomal protein P0 *Rhipicephalus microplus* [AGQ49465.1]	1^e-29^
GBAK01000283	BP13-14	ribosomal protein S17 *Dermacentor variabilis* [ACF35534.1]	3^e-59^
		ribosomal protein S17 *Argas monolakensis* [ABI52710.1]	1^e-55^
		ribosomal protein S17, partial *Ixodes scapularis* [AAY66942.1]	2^e-55^
GBAI01000085	BP6	ribosomal protein S19, putative *Ixodes scapularis* [XP_002408352.1]	1^e-38^
GBAJ01000004	BP10		2^e-25^
GBAK01000158	BP13-14		2^e-97^
GBAI01000070	BP6	ribosomal protein S20, putative *Ixodes scapularis* [XP_002405144.1]	9^e-58^
		ribosomal protein S20 *Argas monolakensis* [ABI52775.1]	8^e-58^
GBAJ01000011	BP10	ribosomal protein S20, putative *Ixodes scapularis* [XP_002405144.1]	5^e-59^
		ribosomal protein S20 *Argas monolakensis* [ABI52775.1]	3^e-59^
GBAK01000380	BP13-14	ribosomal protein S20, putative *Ixodes scapularis* [XP_002405144.1]	1^e-52^
		ribosomal protein S20 *Argas monolakensis* [ABI52775.1]	2^e-51^
GBAI01000045	BP6	40S ribosomal protein S21 *Ixodes scapularis* [Q4PM64.1]	9^e-45^
GBAJ01000020	BP10		1^e-51^
GBAL01000117	BP15-16		7^e-44^
GBAI01000103	BP6	40S ribosomal protein S23 *Ixodes scapularis* [Q86FP7.1]	9^e-60^
GBAI01000056	BP6	ribosomal protein S24 *Ixodes scapularis* [AAY66904.1]	2^e-66^
GBAJ01000083	BP10		4^e-69^
GBAK01000149	BP13-14	ribosomal protein S24 *Dermacentor variabilis* [AAY40467.1]	2^e-79^
GBAL01000012	BP15-16		5^e-77^
GBAK01000274	BP13-14	ribosomal protein S25 *Ixodes scapularis* [AAY66882.1]	5^e-47^
GBAK01000217	BP13-14	ubiquitin/ribosomal protein S27a fusion protein *Dermacentor variabilis* [ACF35544.1]	1^e-79^
GBAL01000172	BP15-16		8^e-32^
GBAL01000116	BP15-16	ubiquitin/40S ribosomal protein S27a *Ornithodoros parkeri* [ABR23473.1]	6^e-43^
GBAK01000021	BP13-14	40S ribosomal protein S27 *Ixodes scapularis* [AAY66945.1]	2^e-44^
GBAL01000206	BP15-16		3^e-28^
GBAL01000058	BP15-16	40S ribosomal protein S28 *Ornithodoros parkeri* [ABR23349.1]	4^e-25^
GBAI01000066	BP6	40S ribosomal protein S30 *Ixodes scapularis* [AAY66965.1]	2^e-44^
GBAK01000194	BP13-14		3^e-65^
GBAL01000160	BP15-16		4^e-65^
GBAK01000279	BP13-14	60S acidic ribosomal protein P1, putative *Ixodes scapularis* [XP_002435967.1]	3^e-32^
GBAK01000387	BP13-14	20S proteasome, regulatory subunit beta, putative *Ixodes scapularis* [XP_002406585.1]	1^e-79^
GBAI01000100	BP6	Mitochondrion 16S ribosomal RNA (16S rRNA) gene *Amblyomma americanum* [L34313.1]	3^e-42^
GBAK01000003	BP13-14		3^e-68^
GBAL01000010	BP15-16		3^e-68^

**Table 8:** **Putative immunogenic proteins of miscellaneous function in** ***Amblyomma americanum*** **tick saliva**

Accession#	Source library	Top matches in GenBank [accession#]	e-values
GBAK01000340	BP13-14	transmembrane protein, putative *Ixodes scapularis* [XP_002406433.1]	7^e-30^
GBAK01000341	BP13-14		1^e-12^
GBAK01000135	BP13-14	signal sequence receptor beta *Ixodes scapularis* [XP_002414113.1]	5^e-108^
GBAL01000069	BP15-16		2^e-85^
GBAK01000145	BP13-14	selenoprotein, putative *Ixodes scapularis* [XP_002400767.1]	1^e-11^
GBAK01000160	BP13-14	selenoprotein K, putative *Ixodes scapularis* [XP_002403087.1]	1^e-06^
		selenoprotein K *Amblyomma variegatum* [DAA34408.1]	2^e-06^
GBAI01000072	BP6	salivary selenoprotein M precursor *Ixodes scapularis* [AAY66722.1]	1^e-29^
GBAK01000292	BP13-14	beta-actin mRNA, complete cds *Dermacentor variabilis* [EF488512.2]	4^e-13^
GBAK01000044	BP13-14	myosin heavy chain, skeletal muscle or cardiac muscle, putative *Ixodes scapularis* [XP_002433460.1]	8^e-60^
GBAI01000046	BP6	myosin alkali light chain protein *Haemaphysalis longicornis* [ADN34300.1]	2^e-41^
GBAJ01000045	BP10		3^e-35^
GBAJ01000066	BP10		2^e-39^
GBAK01000412	BP13-14	myosin light chain 1, putative *Ixodes scapularis* [XP_002414092.1]	1^e-31^
GBAK01000014	BP13-14	nonmuscle myosin essential light chain *Ixodes scapularis* [XP_002407055.1]	1^e-97^
GBAK01000264	BP13-14	paramyosin *Haemaphysalis longicornis* [AFR32950.1]	3^e-45^
GBAI01000090	BP6	beta-tubulin, putative *Ixodes scapularis* [XP_002403010.1]	3^e-10^
GBAK01000075	BP13-14	dynein light chain *Ixodes scapularis* [XP_002408929.1]	1^e-60^
		dynein light chain type 1 *Rhipicephalus microplus* [AHH29554.1]	2^e-60^
GBAJ01000053	BP10	zinc finger protein, putative *Ixodes scapularis* [XP_002413941.1]	2^e-81^
GBAK01000084	BP13-14	zinc finger protein, putative *Ixodes scapularis* [XP_002406104.1]	1^e-28^
GBAL01000027	BP15-16		8^e-29^
GBAL01000011	BP15-16	zinc finger protein, putative *Ixodes scapularis* [XP_002408238.1]	1^e-06^
GBAI01000010	BP6	KSR 2 misexpression suppressor *Amblyomma variegatum* [DAA34564.1]	7^e-42^
GBAK01000152	BP13-14		3^e-42^
GBAL01000052	BP15-16		3^e-42^
GBAK01000081	BP13-14	Misexpression suppressor of KSR, putative, mRNA *Ixodes scapularis* [XM_002411057.1]	6^e-11^
GBAK01000363	BP13-14	BRI1-KD interacting protein, putative *Ixodes scapularis* [XP_002434447.1]	2^e-07^
GBAL01000026	BP15-16	histone H2 *Dermacentor variabilis* [ACF35543.1]	2^e-66^
GBAL01000127	BP15-16	DEK domain-containing protein, putative *Ixodes scapularis* [XP_002400977.1]	3^e-23^
GBAK01000039	BP13-14	small nuclear ribonucleoprotein sm D2, putative *Ixodes scapularis* [XP_002411748.1]	2^e-62^
GBAL01000037	BP15-16		9^e-63^
GBAK01000113	BP13-14	snrnp sm protein, putative *Ixodes scapularis* [XP_002410602.1]	7^e-05^
GBAL01000243	BP15-16	translation initiation factor 3 and TGF-beta interacting protein, putative *Ixodes scapularis* [XP_002400973.1]	1^e-54^
GBAI01000025	BP6	translation initiation factor eIF3, p35 subunit, putative *Ixodes scapularis* [XP_002412399.1]	1^e-57^
GBAL01000007	BP15-16	translation initiation factor 4 F cap-binding subunit *Amblyomma variegatum* [DAA34255.1]	3^e-05^
GBAL01000135	BP15-16	translation factor *Amblyomma variegatum* [DAA34728.1]	4^e-22^
GBAJ01000048	BP10	eIF2B-gamma protein, putative *Ixodes scapularis* [XP_002414374.1]	4^e-39^
GBAK01000330	BP13-14	translation initiation factor 4 F, helicase subunit, putative *Ixodes scapularis* [XP_002407236.1]	1^e-81^
		DEAD box ATP-dependent RNA helicase, putative *Ixodes scapularis* [XP_002414033.1]	2^e-69^
GBAL01000220	BP15-16	ribosomal DEAD box protein, putative *Ixodes scapularis* [XP_002414143.1]	3^e-126^
		DEAD box ATP-dependent RNA helicase, putative *Ixodes scapularis* [XP_002414033.1]	8^e-62^
GBAJ01000025	BP10	ATP-dependent RNA helicase pitchoune, putative *Ixodes scapularis* [XP_002401339.1]	5^e-128^
GBAK01000055	BP13-14	translation elongation factor EF-1 alpha/Tu, putative *Ixodes scapularis* [XP_002411147.1]	5^e-45^
GBAK01000396	BP13-14	elongation factor 1 gamma, putative *Ixodes scapularis* [XP_002410199.1]	5^e-12^
GBAK01000102	BP13-14	isolate RAHD_87 Ruka SINE elements *Rhipicephalus appendiculatus* [EU018131.1]	3^e-16^
GBAI01000020	BP6	transcription initiation factor IIA gamma chain, putative *Ixodes scapularis* [XP_002410024.1]	1^e-69^
GBAK01000153	BP13-14	transcription initiation factor IID subunit *Argas monolakensis* [ABI52792.1]	5^e-27^
GBAK01000297	BP13-14		1^e-18^
GBAL01000213	BP15-16	transcription factor E2F7, putative *Ixodes scapularis* [XP_002401356.1]	6^e-06^
GBAL01000259	BP15-16	transcription initiation factor TFII-D, subunit TAF10, putative *Ixodes scapularis* [XP_002409390.1]	7^e-09^
GBAK01000321	BP13-14	transcription factor containing NAC and TS-N domains, putative *Ixodes scapularis* [XP_002413138.1]	1^e-17^
		transcription factor *Amblyomma variegatum* [DAA34590.1]	4^e-12^
GBAK01000162	BP13-14	methyl-CpG binding transcription regulator, putative *Ixodes scapularis* [XP_002407962.1]	3^e-12^
GBAK01000056	BP13-14	cleavage/polyadenylation factor Ia subunit Clp1p, putative *Ixodes scapularis* [XP_002406587.1]	3^e-94^
GBAK01000239	BP13-14	z-protein, putative *Ixodes scapularis* [XP_002401042.1]	6^e-20^
GBAK01000270	BP13-14		5^e-20^
GBAK01000212	BP13-14	coiled-coil domain-containing protein, putative *Ixodes scapularis* [XP_002402814.1]	1^e-32^
GBAL01000077	BP15-16		9^e-33^
GBAL01000049	BP15-16	activating signal cointegrator 1 complex subunit, putative *Ixodes scapularis* [XP_002401442.1]	8^e-43^
GBAL01000076	BP15-16		2^e-16^
GBAK01000231	BP13-14	cytochrome C, putative *Ixodes scapularis* [XP_002411082.1]	2^e-54^
GBAJ01000096	BP10	cytochrome C *Dermacentor variabilis* [AAY86487.1]	2^e-62^
GBAJ01000064	BP10	histamine release factor mRNA, complete cds *Boophilus microplus* [DQ009479.1]	2^e-62^
		histamine release factor mRNA, complete cds *Amblyomma americanum* [DQ009481.1]	1^e-09^
		IgE-dependent histamine release factor mRNA, complete cds *Dermacentor variabilis* [AF467699.1]	2^e-09^
		histamine release factor mRNA, complete cds *Dermacentor andersoni* [DQ009480.1]	2^e-07^
GBAK01000197	BP13-14	histamine release factor mRNA, complete cds *Boophilus microplus* [DQ009479.1]	2^e-43^
		histamine release factor mRNA, complete cds *Amblyomma americanum* [DQ009481.1]	3^e-19^
		IgE-dependent histamine release factor mRNA, complete cds *Dermacentor variabilis* [AF467699.1]	5^e-23^
		histamine release factor mRNA, complete cds *Dermacentor andersoni* [DQ009480.1]	1^e-23^
GBAK01000077	BP13-14	amercin *Amblyomma americanum* [ABI74752.1]	1^e-36^
		persulcatusin *Ixodes persulcatus* [BAH09304.1]	4^e-21^
		defensin 2 *Haemaphysalis longicornis* [ABW08118.1]	1^e-20^
GBAK01000137	BP13-14	putative beta thymosin *Dermacentor variabilis* [AAO92284.1]	8^e-36^
GBAK01000092	BP13-14	gephyrin, putative *Ixodes scapularis* [XP_002404228.1]	1^e-67^
GBAK01000219	BP13-14	SNAP-25 (synaptosome-associated protein) component of SNARE complex, putative *Ixodes scapularis* [XP_002404177.1]	9^e-46^
GBAL01000092	BP15-16	vAMP-7, putative *Ixodes scapularis* [XP_002400270.1]	7^e-50^
GBAJ01000033	BP10	metaxin, putative *Ixodes scapularis* [XP_002404066.1]	2^e-131^
GBAK01000335	BP13-14	chaperonin complex component, TCP-1 eta subunit, putative *Ixodes scapularis* [XP_002415178.1]	3^e-07^
GBAK01000037	BP13-14	fidipidine, putative *Ixodes scapularis* [XP_002416086.1]	1^e-06^
GBAJ01000072	BP10	tetraspanin-like protein *Dermacentor variabilis* [AAL75584.1]	1^e-87^
GBAI01000038	BP6	saposin, putative *Ixodes scapularis* [XP_002412058.1]	6^e-54^
GBAL01000086	BP15-16	UBX domain-containing protein, putative *Ixodes scapularis* [XP_002413353.1]	0.004
GBAK01000033	BP13-14	UBX domain-containing protein, putative *Ixodes scapularis* [XP_002403277.1]	2^e-09^
GBAK01000302	BP13-14	MYC-induced nuclear antigen, putative *Ixodes scapularis* [XP_002434908.1]	1^e-11^
GBAL01000246	BP15-16	mitotic spindle assembly checkpoint protein MAD2A, putative *Ixodes scapularis* [XP_002401838.1]	1^e-40^
GBAK01000377	BP13-14	tumor rejection antigen (gp96), putative *Ixodes scapularis* [XP_002413149.1]	8^e-61^
		Hsp90 protein, putative *Ixodes scapularis* [XP_002414808.1]	3^e-18^
GBAJ01000015	BP10	heat shock 70 kDa protein 5 *Haemaphysalis longicornis* [ACA84007.1]	5^e-13^
		heat shock protein, putative *Ixodes scapularis* [XP_002433656.1]	4^e-10^
GBAL01000001	BP15-16	heat shock 70 kDa protein 5 *Haemaphysalis longicornis* [ACA84007.1]	2^e-50^
		heat shock protein, putative *Ixodes scapularis* [XP_002433656.1]	8^e-41^

### Orphan immunogenic tick saliva protein coding cDNAs

Table 2 lists 86 contig sequences that code for orphan immunogenic *A. americanum* tick saliva proteins that show high amino acid identity with other tick proteins, but not with non-tick organisms. Of the 86 sequences, 12 sequences each were identified in BP6 and BP10, while 34 and 28 sequences were identified in BP13-14 and BP15-16, respectively. It is notable that except for 23 sequences that show similarity exclusively to other metastriata tick proteins, the remaining 63 sequences show similarity either exclusively to *Ixodes* spp. ticks or to both metastriata and prostriata tick proteins. In general metastriata and prostriata tick protein sequences tend to show low amino acid conservation. Thus, the 63 sequences in Table 2 that show high conservation could represent important immunogenic tick saliva proteins that regulate key tick physiological functions. Observations here that majority of *A. americanum* sequences in Table 2 show similarity to prostriata than metastriata tick proteins could be due to that at the time of this write up, there were more *I. scapularis* protein sequence entries in GenBank than metastriata tick entries. Thus some of the sequences that show similarity exclusively to *I. scapularis* might also be conserved in other metastriata ticks if genome sequence data became available.

Some notable observations in Table 2 include the GBAI01000007 sequence, which showed 74-97% amino acid identity with putative immunogenic secreted proteins from saliva of *Rhipicephalus annulatus*, *Haemaphysalis quinghaiensis*, *Haemaphysalis longicornis*, and *I. scapularis* (not shown). Likewise sequence GBAI01000013 showed similarity to mucin-like proteins previously described in *Dermacentor variabilis* and *Amblyomma variegatum* [71, 72]. Mucins are heavily glycosylated proteins with numerous functions including lubrication, cell signaling, and host defense against pathogens [73, 74]. Sequences GBAI01000021, GBAK01000213, and GBAL01000042 match with recently described *A. americanum* secreted saliva protein AV422 [70]. This cross-tick species conserved protein is characterized with 14 conserved cysteine amino acid residues predicted to form 7 disulfide bonds. Mulenga et al. [70] revealed the role of described protein in interrupting host hemostasis and complement activation. It is also interesting to note that a protein that is 97% identical to AV422 was recently described in the *R. microplus* proteome [75]. Additionally, *R. microplus* proteome [75] contains sequences which show a high level of identity to GBAI01000007, GBAI01000051, GBAK01000417, GBAK01000324, and GBAK01000378 sequences related to immunogenic hypothetical *A. americanum* proteins. Highly conserved proteins such as AV422, and others described here could represent important target antigens for the development of universal anti-tick vaccines, which are highly advocated for [30]. Male biopanned library originating sequence GBAL01000253 showed 80% amino acid sequence identity with putative *I. scapularis* 65 kDa macrophage protein. The role(s) of its intracellular mammalian ortholog is poorly understood, but a predicted phosphorylation site and a series of Ca^2+^ binding domains indicate that this protein could be involved in processes determining macrophage activity in immune response [76].

### *A. americanum*tick saliva immunogenic proteases

Proteases are central to the physiology of all organisms. In tick physiology, proteases were linked to embryo development [77, 78], blood meal feeding [79], and host blood processing in the midgut [80]. Interfering with tick protease function, as revealed by RNAi silencing [81, 82], and feeding of ticks on animals immunized with recombinant proteases [83], affected tick feeding efficiency and reproduction. Here for the first time, we indicate that some protease-like molecules including Leukotriene A-4 hydrolase (LTA4H), asparaginyl endopeptidase, cysteine proteinases, carboxy- and metallo-proteases, as well as ubiquitin fusion degradation protein, are present in *A. americanum* tick saliva at the beginning of the tick feeding process (Table 3).

The presence of LTA4H in *A. americanum* is interesting and counter-intuitive. In mammals, the LTA4H enzyme catalyzes the last step in biosynthesis of leukotriene B4 (LTB4), a potent chemo attractant and proinflammatory lipid mediator derived from arachidonic acid [84, 85] that is involved in immune responses [86], host defense against infection [87], platelet activation [88], and lipid metabolism [89]. The pro-host defense functions of LTA4H are contrary to what the tick is expected to do to accomplish feeding, to block inflammation and other host defense mechanisms. From this perspective, it will be interesting to investigate if *A. americanum* LTA4H is functional. There is a possibility that *A. americanum* LTA4H performs other functions at the tick-feeding site.

Of the 18 sequences in Table 3, six sequences GBAI01000024, GBAJ01000008, GBAK01000094, GBAK01000180, GBAK01000182 and GBAK01000183 are provisionally identified as cathepsin-like cysteine proteases. Accumulating evidence suggests that secretion of cysteine proteases in *A. americanum* tick saliva is relatable to tick and host interactions. In mammals, cathepsin B and L are ubiquitously expressed and multifunctional, intracellular and extracellular [90]. They are involved with protein turnover housekeeping function in lysosomes [91], degradation of extracellular matrix [92], elimination of cellular mediators of inflammation such as neutrophils [93], and recent studies have demonstrated extracellular activity of human cathepsin B and L associated with inflammation function [94]. Ticks are pool feeders that accomplish feeding by disrupting host tissue and sucking up the blood that bleeds into the wound [95]. This feeding style is expected to provoke host tissue repair response, to which inflammation and extracellular matrix modeling are important [96, 97]. Thus, if functional, it is conceivable that tick saliva cathepsin B and L-like cysteine proteases could speculatively disrupt tissue repair response by killing cellular mediators of inflammation and destroying the extracellular matrix. There is indirect evidence for other parasites that show cysteine proteases as major components of the parasitic excretory-secretory immuno-proteome in *Spirometra mansoni* [98] and *Euclinostomum heterostomum* [99]. In related studies *Fasciola hepatica* secretes a cathepsin-L protease linked to invasion of host tissue by this parasite [100], while application of cysteine protease inhibitors had anti-parasitic effects against Chagas’ disease [101] and malaria [102] parasites. A lone study showed that *H. longicornis* cathepsin-L like cysteine protease, longipain displayed babesiacidal activity via specific adherence to the parasite membranes [103], which could indicate a role for cysteine proteases in tick immunity. Recently, *R. microplus* proteome analysis [75] revealed the presence of cathepsin-like cysteine protease sequence which appeared similar to GBAK01000094, GBAK01000182 and GBAK01000183 sequences described in this study.

In Table 3 we also listed sequence GBAK01000372, which showed identity to putative legumain-like protease precursor in *D. variabilis*. Legumain is a lysosomal cysteine protease that has a strict specificity for hydrolysis of asparaginyl bonds [104]. In human trematode parasite of blood vessels *Schistosoma mansoni*, this protease plays an important role in host hemoglobin degradation to diffusible peptides [105]. The same role is predicted for tick legumains found in the gut of *H. longicornis* [106] and *Ixodes ricinus* [107], as well as in sialotranscriptome analyses of *Amblyomma maculatum* [108].

Three sequences in Table 3, GBAJ01000040, GBAK01000214, and GBAL01000134 are provisionally identified as serine carboxypeptidases. Very little is known on serine carboxypeptidases in tick physiology research. A lone study characterized a *H. longicornis* tick serine carboxypeptidase that is predominantly expressed in midgut and up regulated in response to tick feeding with the yeast expressed recombinant protein cleaving substrates similar to those of human serine carboxypeptidase cathepsin A [109]. Indirect evidence in helminth parasites, *Strongyloides ratti* [110] and *Angiostrongylus cantonensis* [111], excretory-secretory proteomes suggests that serine carboxypeptidases are part of the protein complex that regulates helminth parasite and host interactions. The presence of serine carboxypeptidases in *A. americanum* tick saliva may indicate that similar to other parasites, ticks use this class of proteins to regulate tick-host interactions. It is notable that cathepsin A, the mammalian serine carboxypeptidases, has been linked to vasodilation, with malfunctions or deficiencies of this protein causing vasoconstriction [112]. One of the host’s defense mechanisms to the tick feeding style of lacerating host tissue is vasoconstriction. Could tick saliva serine carboxypeptidase be involved in mediating the tick’s vasodilating function? On the other hand, sequence GBAK01000358 shows identity to angiotensin-converting enzyme, a carboxypeptidase responsible for converting angiotensin I to angiotensin II, which constricts the blood vessels [113].

Four of the 18 sequences in Table 3, GBAK01000026, GBAK01000111, GBAK01000132, and GBAK01000196 show identity to annotated tick metalloproteases, with the last sequence showing identity to *I. scapularis* endothelin-converting enzyme. Expression of metalloproteases in tick salivary glands has been widely reported [114–116], with RNAi silencing of some affecting tick feeding efficiency and fertility in *R. microplus* [117] and *I. ricinus* [118], while feeding ticks on rabbits immunized with a recombinant tick metalloprotease affected *H. longicornis* tick feeding efficiency [119]. In a lone study, an *I. scapularis* recombinant metalloprotease similar to reprolysin had gelatinase and fibrinolytic activities [120]. Preventing blood clotting is among the most important “must do’s” for ticks to successfully feed. From this perspective it is possible that the four metalloproteases found in *A. americanum* tick saliva could play important roles in facilitating tick feeding. It is also interesting to note that excretory-secretory proteomes of several blood feeding and/or dwelling parasites such as *Haemonchus contortus* [121] *, Onchocerca volvulus* [122], *A. cantonensis* [123, 124], *Ancylostoma caninum* [125], *Ancylostoma ceylanicum* [126], and *Clonorchis sinensis* [127] have metalloproteases, which regulate interactions of these parasites with their vertebrate hosts. It is also notable that metalloproteases are a major component of snake venom that is responsible for hemorrhage and may also interfere with the hemostatic system [128]. The occurrence of an endothelin-converting enzyme-like protein in tick saliva could be considered counter-intuitive in that in mammals this protease is involved in proteolytic processing of endothelins, which are potent vasoconstrictor molecules [129]. Ticks feed over a long period of time, and thus to continue feeding, host blood vessels must stay dilated. With the help of endothelins, the host constricts its blood vessels in response to injury as occurs during tick feeding to prevent further blood loss. The tick overcomes this defense by secreting vasodilator molecules into the feeding site. From this perspective the observation of an endothelin-converting enzyme-like protein in *A. americanum* was surprising.

A lone sequence, GBAK01000269 showed similarity to ubiquitin fusion-degradation protein, a protease, which is involved in degradation of ubiquitin tagged proteins [130]. Apart from the annotated *I. scapularis* sequence in GenBank, the ubiquitin fusion-degradation protein appears to have not been studied prior to this report. Ubiquitin and ubiquitin fusion-degradation protein are part of the protein clearance system. It will be interesting to investigate if the ubiquitin fusion-degradation protein observed is functional. If so, could it be used by ticks to prematurely clear host defense factors? It is interesting to note that in addition to ubiquitin fusion-degradation protein in Table 3, we also observed that *A. americanum* secretes ubiquitin ligase as well as ubiquitin/ribosomal protein S27a fusion protein (Tables 6 and 7) during feeding. Could it be that the tick uses its own ubiquitin and ubiquitin fusion-degradation protein to tag and trigger degradation of host defense factors?

### Protease inhibitors

Secretion of protease inhibitors in tick saliva as observed is not surprising. Given that host defenses against parasites are predominantly mediated by proteases, it’s widely hypothesized in parasite and host interaction studies that parasites including ticks could utilize protease inhibitors to evade host defenses [131, 132]. Table 4 lists 19 sequences that have been provisionally identified as inhibitors, serine protease inhibitors (GBAI01000043, GBAI01000058, GBAJ01000023, GBAJ01000027, GBAJ01000043, GBAK01000040, GBAK01000091, GBAK01000277, GBAK01000097, GBAL01000201 and GBAL01000059), cysteine protease inhibitors (GBAK01000064, GBAL01000013, and GBAL01000180), tick carboxypeptidase inhibitor (GBAK01000073), ATPase inhibitor (GBAJ01000071 and GBAK01000345), and translation initiation inhibitor (GBAK01000027 and GBAL01000098). It is notable, but not surprising that more than half of inhibitors found in *A. americanum* saliva in this study are putative inhibitors of serine proteases. Serine proteases have the “lions share” as mediators of the host’s defense pathway to tick feeding [132], and thus it is conceivable that the majority of inhibitors in tick saliva will target serine proteases. It is interesting to note that the majority of reported tick salivary gland or tick saliva protease inhibitors inhibit serine proteases or serine protease mediated pathways including elastase [52, 133, 134], trypsin [52, 134, 135], thrombin [135], factor Xa [135], blood clotting in general [52, 135], and complement activation [52, 136]. Mammalian cysteine proteases play important roles in mediating host defense reactions [132, 137], and thus it is logical that *A. americanum* may secrete cystatins, the cysteine protease inhibitors in its saliva. One of the three cystatins, GBAL01000180 show high amino acid identity to *R. microplus* cystatin (AGW80658.1), a validated functional inhibitor of mammalian cysteine proteases [138]. Likewise the tick carboxypeptidase inhibitor found in *A. americanum* tick saliva shows high amino acid identity to a functionally characterized homolog in *R. bursa,* which has fibrinolytic function suggesting that it plays roles in blood meal feeding [139]. Nucleotides ADP, ATP, and UTP released into the extracellular environment play roles in inflammation [140], and are powerful chemotactic stimuli for immune response cells [141], functions that must be blocked for the tick to successfully feed. There is a possibility that the ATPase inhibitor found in *A. americanum* tick saliva could participate in blocking extracellular ATP function and in so doing, allow the tick to evade host defense reactions. The occurrence of the translation initiation inhibitor in *A. americanum* tick saliva is intriguing. The general function of the translation initiation inhibitor is to disrupt synthesis of new proteins. The most immediate question is to test whether or not tick translation initiation inhibitor is functional, and if so does it internalize into host cells? If so does it block synthesis of new proteins at the tick-feeding site?

### *A. americanum*tick saliva transporters and/or ligand binding proteins

We have provisionally identified *A.americanum* tick saliva transporters and/or binding proteins in Table 5 based on their putative ligand: iron and heme, calcium, immunoglobulin G, histamine, lipid and and/or fatty acid, actin, nucleic acid, insulin and miscellaneous. Except for proteins in the miscellaneous group, the occurrences of the majority of proteins in Table 5 are relatable to suspected molecular interactions between the host and the tick.

### Ferritin and hemelipoprotein

Six and three sequences are provisionally identified as ferritin (GBAI01000078, GBAJ01000019, GBAJ01000036, GBAJ01000098, GBAK01000111, and GBAL01000165) and hemelipoglycoprotein (HeLp) (GBAJ01000035, GBAK01000315, and GBAL01000006), respectively. Both ferritin [142–145] and HeLp [146, 147] have been cloned and characterized in multiple tick species. During feeding and blood meal processing ticks are faced with the high risk of oxidative stress because of huge amounts of iron in host blood, and heme, a bi-product of hemoglobin digestion [148]. Although mechanisms need further clarification, ticks are protected against iron and heme mediated oxidative stress by ferritin [149] and HeLP [150]. Hajdusek et al. [149] proposed that ferritin bound host blood-derived iron, while HeLp bound heme and delivered it to tick tissues to meet cell requirements. The observation in this study that ticks inject ferritin and HeLp into the host during tick feeding raises an interesting question of whether or not the tick utilizes tick saliva ferritin and HeLp to dump iron and heme into the host to avoid oxidative stress. Tick secretion of ferritin and HeLp into the feeding-site could also benefit tick borne disease agents that may need iron and/or heme to proliferate [151, 152]. Except for microbial organisms such as *Borrelia burgdorferi*, which do not require iron for growth [153], most microbial organisms need iron and/or heme to proliferate [154, 155]. As an anti-microbial defense, mammalian hosts utilize ferritin to sequester iron and deny microbial organisms’ access to it [156]. From this perspective, it is logical to speculate that presence of ferritin and HeLp in tick saliva is advantageous to microbial organisms in that secreted ferritin and HeLp will deliver iron and heme into the feeding site to the advantage of the transmitted tick borne disease agent. It interesting to note that ferritin was up regulated in *D. variabilis* ticks that were infected with *Rickettsia montana* [157].

Data in this study also advance our knowledge on the biology of tick ferritins. Two ferritin cDNAs have been described in ticks, ferritin-1 which is deemed intracellular because it does not have a signal peptide, and ferritin-2, deemed secreted because it has a signal peptide [142, 149]. It is notable that both of the two-ferritin sequences (GBAI01000078 and GBAJ01000019) in this study showed high amino acid identity to ferritin 1 and not ferritin 2 (not shown). The partial ferritin sequence fragment in this study, GBAI01000078 is 100% identical to *A. americanum* ferritin-1 (AAQ54708.1). In contrast, ferritin sequence GBAJ01000019 showed ~60% identity to tick ferritin-1 sequences and 98% amino acid identity to mammalian ferritin such as *Canis lupus familiaris* (NP_001003080.1) (not shown). Although further verification is needed data presented here indicates the presence of a third ferritin in ticks. It is also interesting to note that similar to dog ferritin-1 (NP_001003080.1), which is 98% identical to GBAJ01000019 does not have a signal peptide. These data may suggest that ferritin in *A. americanum* tick saliva are secreted in a non-canonical way. Consistent with findings in this study, tick ferritin and HeLp were detected in partially and fully engorged *R. microplus* proteome [75].

### Calcium binding proteins

Table 5 lists sequences showing identity to four putative calcium binding proteins, including calmodulin (GBAI01000083, GBAJ01000110, GBAK01000031, and GBAL01000053), calponin (GBAI01000101), calreticulin (GBAJ01000101 and GBAK01000025), and sarcoplasmic calcium binding protein (GBAL01000044). To complete feeding ticks must keep host blood in a fluid state at the tick-feeding site and in the midgut. Thus, given that calcium (Ca^2+^) is the fourth co-factor of the blood clotting activation pathway [158], it is conceivable that ticks may secrete Ca^2+^ binding proteins into the tick-feeding site to bind and deplete Ca^2+^ and prevent activation of blood clotting. Apart from potential Ca^2+^ function, the four *A. americanum* tick saliva putative Ca^2+^ binding proteins in Table 5 could perform multiple other yet unknown functions in tick physiology. In relative terms, calreticultin (CRT) is the most studied tick calcium-binding protein; it is a validated cross-tick species conserved immunogenic tick saliva protein [159] that is currently used as a biomarker for human tick bites [160]. However beyond this, the role(s) of CRT at the tick-feeding site are unknown. Recombinant CRT of parasites such as *Trypanosoma cruzi* [161], *H. contortus* [162], *Entamoeba histolytica* [163], *Trypanosoma carassii* [164], blocks the complement activation cascade by binding C1q, the first factor of the cascade. Additionally *H. contortus* calreticulin was also shown to bind factor Xa [162], an important protease of the blood-clotting cascade [165]. In a related study (Kim and Mulenga, *unpublished*), showed that a yeast expressed recombinant *A. americanum* calreticulin bound C1q, but did not interfere with complement activation. From these data tick calreticulin may function differently when compared to other parasites. It is also interesting to note that both mammalian and parasite calreticulin were shown to enhance wound healing [166]. Whether or not, tick saliva calreticulin enhances wound healing has not been reported. If consistent with observations in other organisms, it would be counter-intuitive for tick calreticulin to enhance wound healing. Ticks begin the feeding process by creating a feeding lesion, and to continue feeding, ticks must block wound healing mechanisms for the feeding site to remain viable [167]. Similar to calreticulin, calmodulin is multifunctional protein that is primarily known for its roles in Ca^2+^ homeostasis in mammals [168]. Except for a single study that described a *H. longicornis* tick calmodulin-like protein [169], nothing is known about this protein in tick physiology. Similarly, there are no studies on the roles of calponin in tick physiology. In mammals, calponin performs multiple functions including regulation of actin, Ca^2+^, and ATPase [170–172]. Whether or not tick calponin is multi-functional remains to be determined. Similarly, there are no reported studies on the sarcoplasmic-calcium binding protein in ticks. This molecule has been characterized in multiple invertebrates [173–175], and is considered a key factor in human allergic reactions to shrimp [174, 175]. It will be interesting to find out what these proteins do at the tick-feeding site.

### Fatty acid and histamine (lipocalin) binding proteins

Eight sequences (GBAI01000035, GBAK01000066, GBAK01000093, GBAK01000144, GBAK01000151, GBAL01000122, GBAL01000248, and GBAL01000133) and one sequence (GBAJ01000056) are provisionally identified as putative histamine binding/lipocalin and fatty acid binding protein, respectively. Presence of lipid derivatives in tick saliva has been demonstrated, and been shown to play important roles in tick feeding success. Prostaglandins (PGs), derivatives of arachidonic acid, are important vasodilators, which contributes to the tick’s ability to block vasoconstriction of host blood vessels as a host defense response to tissue injury during tick feeding [176]. Several studies demonstrated secretion of PGE2 [177–179] and prostacyclin (PGI) [32] in tick saliva. Tick saliva PGE2 was shown to interfere with function of macrophages [180] and dendritic [181] cells, which are important in host defense response [182, 183]. Despite the demonstration that PGs are secreted into tick saliva, their mode of secretion has not been elucidated. There is a possibility that lipid-binding proteins found in this study serve as PG transporters. We would like to caution here that *A. americanum* hemolymph PGE2 was found not bound to any carrier protein [35] suggesting that it may not require a transporter protein. Thus there is a possibility that putative lipid binding proteins described here may perform other functions at the tick feeding site that may not be related to transporting lipids from the tick into the tick-feeding site.

Ticks have to overcome the host’s inflammation response to complete feeding. Histamine is a potent pro-inflammatory molecule that is released by cellular mediators of inflammation such as mast cells and neutrophils [184]. Thus, it is logical that *A. americanum* tick saliva contains proteins like histamine-binding proteins/lipocalin to sequester histamine and stop the inflammation response. Histamine mediated cutaneous inflammation is one the host’s defense reactions to tick feeding, as demonstrated by adverse effects on tick attachment, feeding efficiency, and reproductive success when histamine was elevated at the feeding site [185, 186].

### Nucleic acid binding proteins

Table 5 lists three putative nucleotide binding proteins, GBAK01000068, GBAL01000186, and GBAJ01000103 that have been provisionally identified as respective GTP-binding, ATP-binding, and histidine triad known as purine-binding protein [187]. In addition, four sequences related to RNA- (GBAL01000055, GBAL01000072, and GBAL01000222) and DNA- (GBAK01000395) binding proteins were identified. Tick feeding involves disrupting host cells, which could lead to release of nucleic acids outside the cell. Extracellular nucleic acids are potent pro-inflammatory molecules. Thus listed putative nucleic acid binding proteins could be part of the ticks system to modulate host inflammation response to tick feeding activity. Here it is appropriate to mention that a number of sequences which showed identity to different transcriptional and translational factors, which are predicted to interact with nucleic acids (GBAI01000020, GBAI01000025, GBAJ01000025, GBAJ01000048, GBAK01000039, GBAK01000055, GBAK01000102, GBAK01000113, GBAK01000153, GBAK01000162, GBAK01000297, GBAK01000321, GBAK01000330, GBAK01000337, GBAL01000007, GBAL01000037, GBAL01000127, GBAL01000135, GBAL01000213, GBAL01000220, GBAL01000243, and GBAL01000259), are listed in the Table 8.

### Immunoglobulin and other miscellaneous binding proteins

Secretion of immunoglobulin G (IgG) in tick saliva was demonstrated [188], and thus the occurrence of IgG binding protein in *A. americanum* (GBAK01000082 and GBAK01000159) saliva is not surprising. While the possibility that this protein performing other functions at the tick-feeding site cannot be ruled out, it’s most likely that the IgG binding protein in *A. americanum* tick saliva is used to eliminate host antibodies from the tick body during the feeding process, as previously supposed [188, 189].

Sequence GBAK01000246 showed similarity to insulin-like growth factor binding protein-related protein 6 (IGFBP-rP6). Mulenga and Khumthong [190] characterized two alternative IGFBP-rP6 sequences in *A. americanum*, short and long, and using dual RNAi silencing showed reduction in feeding efficiency in treated females. Although the function of IGFBP-rP6 in the tick feeding process is still unclear, our results here confirm the presence of this protein in tick saliva and that it’s antigenic.

A lone sequence GBAL01000113 appeared similar to the chemokine binding protein evasin-1 from *Rhipicephalus sanguineus*, which displayed a high affinity for pro-inflammatory CCL3, CCL4, and CCL18 chemokines [191]. Pro-inflammatory chemokines are responsible for migration of lymphocytes to an injured site, which represents a key event in an immune response. Chemokines CCL3 and CCL4 both attract mononuclear cells, while CCL18 may be involved early in an immune response since it attracts naive CD45RA+ T cells [192]. Chemokine sequestration by a chemokine binding proteins, secreted at the feeding site, helps ticks to evade host immune response.

Table 5 listed two sequences related to cyclophilin A (GBAK01000236 and GBAL01000024), a ubiquitous protein, which binds tightly to potent immunosuppressant cyclosporine A [193] and shows peptidyl-prolyl *cis-trans* isomerase activity [194]. The immunosuppressive action is exerted through complex between cyclophiline A, cyclosporine A, and protein phosphatase known as calcineurin [195]. Phosphatase activity of calcineurin plays a role in activation of T cells [196]. Formation of cyclophiline A and cyclosporine A complex blocks calcineurin phosphatase activity, and subsequently inhibits T cell activation [197]. In addition, recently intracellular cyclophilin A was shown to be an important Ca^2+^ modulator in platelets [198]. Thus, we can speculate that cyclophilin A from tick saliva could be involved in both, evading host immune response and affecting primary hemostasis. It also seems that cyclophilin A plays a role in tick-pathogen interactions. Maeda et al. [199] suggested that cyclophilin A has regulatory role in the growth of *Babesia* parasites in *H. longicornis* ticks.

### Anti-oxidant and other enzymes

Approximately 17% (81/464) of provisionally identified *A. americanum* immunogenic tick saliva protein sequences in this study are associated with anti-oxidant, energy metabolism, and other miscellaneous functions enzymes (Table 6). It is interesting to note that some of the housekeeping enzymes identified in this study were also found in *R. sanguineus* [200] and *R. microplus* [75] tick saliva proteomes ruling out the possibility that observations here could be a result of false antibody binding. In related studies, housekeeping proteins described here, were predicted to be secreted in saliva of *A. americanum* [201], *I. scapularis* [202], *Dermacentor andersoni* [203], and *A. maculatum* [108]. Except for putative anti-oxidants and a few others, the role(s) of the majority of listed enzymes in tick-host interactions are unclear. It is notable, but not surprising that ~50% of *A. americanum* tick saliva enzymes in Table 6 including glutathione-S transferase (GST), protein disulfide isomerase, alkyl hydroperoxide reductase, cytochrome c oxidase, oxidoreductase, gamma-glutamyltransferase, NADH dehydrogenase, thioredoxin reductase, and peroxidase, which are putatively associated with an anti-oxidant functions [204–208]. Injury as occurs at the tick-feeding site induces oxidative stress leading to production of reactive oxygen and nitrogen species (ROS and RNS) as part of the wound healing mechanism [209] and anti-microbial defenses [210]. Several lines of research have shown that many parasites including ticks [211, 212], helminths [213], *Plasmodium* spp. [214], *Trypanosoma* spp. [215], are susceptible to ROS and/or RNS, as revealed by high expression of anti-oxidant enzymes in these parasites or survival of these parasites when anti-oxidant systems of their hosts are impaired [216–218]. Thus it is fitting that *A. americanum* tick saliva will contain such a high number of putative antioxidants. Given the susceptibility of microbial organisms to oxidative stress products, anti-oxidants in tick saliva could be beneficial for transmission of tick borne diseases. It is also interesting to note that given that the tissue destroying effects of oxidative stress products are non-selective, there is a possibility that tick saliva anti-oxidants are protective to host tissue. Tick GST has been studied as a target anti-tick vaccine candidate and seems to confer protection in *R. microplus* and *H. longicornis* [219]. Here it is appropriate to mention that three sequences listed in Table 8 were provisionally identified as selenoproteins K (GBAK01000145 and GBAK01000160) and M (GBAI01000072). Recently, both of them were associated with regulation of cytosolic Ca^2+^ flux, as well as with protective role against oxidative damages [220, 221]. Presence of selenoproteins in saliva increases the power of ticks’ anti-oxidative mechanism. Another notable interesting enzyme is D-dopachrome tautomerase, the functional homolog of macrophage migration inhibitory factor [222], which could be involved in mediating host defense mechanism.

### Ribosomal, heat shock proteins, histamine release factor, and other proteins of miscellaneous functions

Table 7 lists ribosomal proteins in *A. americanum* tick saliva. Although the high number of ribosomal proteins may be surprising, the presence of these proteins in tick saliva is relatable to events that facilitate tick-host interactions. Ribosomal proteins function is predominantly intracellular, but several studies have described extracellular functions of ribosomal proteins in mammals exerting anti-inflammatory activity [223–225]. One of the most studied, ribosomal protein S19 was recently shown to bind the pro-inflammatory cytokines [224]. Whether or not *A. americanum* putative S19 protein (GBAI01000085, GBAJ01000004, and GBAK01000158) will functionally bind pro-inflammatory cytokines remains to be investigated. It is conceivable that ribosomal-like proteins in tick saliva could be part of the tick’s system to evade the host’s inflammation defense response to tick feeding. Indirect evidence suggests that other parasites use ribosomal proteins to evade host defenses. A *Leishmania* S3a ribosomal protein was associated with balancing between Th1 and Th2 immune responses [226], while ribosomal protein L12 was crucial in gonococcal invasion of human reproductive cells [227].

Like ribosomal proteins, extracellular heat shock proteins (HSP) are potent anti-inflammatory molecules [228] and thus, presence of HSP70 and gp96-like proteins in *A. americanum* tick saliva (Table 8) could mean that the tick uses these proteins to evade the host’s inflammation defense against tick feeding. The observation of the tick histamine release factor (tHRF) in *A. americanum* confirms previously published studies that demonstrated presence of functional tHRF in *D. variabilis* tick saliva [62, 63]. What is interesting, however is that the occurrence of this protein in tick saliva is counter-intuitive in that if functional at the tick-feeding site, its actions will promote inflammation, which is what the tick is trying to defeat. It is notable that tHRF was linked to interactions between ticks and pathogens in that the encoding mRNA was up regulated in *R. montana*-infected *D. variabilis* [157] and associated with *Borrelia* transmission by *I. scapularis* ticks [229].

Among other notable proteins of miscellaneous function in *A. americanum* tick saliva (Table 8) include zinc finger-like proteins (GBAJ01000053, GBAK01000084, GBAL01000011, and GBAL01000027). Members of the zinc finger protein family are structurally diverse and are involved with many functions including, replication and repair, transcription and translation, metabolism and signaling, cell proliferation and apoptosis [230]. Typical zinc finger protein ligands include nucleic acids, proteins, and important small molecules [230].

Other interesting sequences include contigs GBAK01000239 and GBAK01000270 that were provisionally identified as z-proteins (Table 8), known as cofactors in coagulation factor Xa degradation by z-protein dependent protease inhibitor [231]. In murine model, z-protein and z-protein dependent protease inhibitor deficiency enhances thrombosis [232], which indicates their important role in preventing blood coagulation in homeostasis. Secretion of tick z-protein at the feeding site probably helps in local inhibition of the host coagulation cascade.

A lone sequence GBAK01000077 showed similarity to defensin proteins, an antimicrobial agent of innate immunity [233]. A defensins were described in several hard tick species. Persulcatusin, from *Ixodes persulcatus*, found to be predominantly expressed in the midgut of adult females, and as recombinant peptide displayed antibacterial activity toward Gram-positive bacteria [234]. Defensin gene in *A. americanum* was found to be expressed in both, midgut and salivary glands [235]. Data here is the first demonstration of defensin proteins in tick saliva. In addition to evading host defenses to tick feeding, and ensuring that blood does not clot, the tick must prevent bacterial or microbial colonization of the feeding site. Thus, it’s conceivable that defensin in tick saliva could be used to keep infections of the feeding site.

Other proteins found in *A. americanum* saliva were related to the protein translation machinery and structural related, myosin-like and paramyosin proteins. The roles of these proteins at the tick-feeding site remain to be explored. It is interesting to note that *R. microplus* paramyosin was reactive with tick immune sera [236, 237] suggesting that it was part of the immunogenic tick saliva protein complex that conferred anti-tick resistance in repeatedly infested animals.

At a glance, it is surprising that we identified a large number of intracellular proteins in tick saliva. We belive this could be due to the mode of salivary secretion. There are described mechanisms of exocytosis of proteins from salivary gland cells into the saliva [238, 239], but massive appearance of originally intracellular proteins in tick saliva suggests actuality of previously proposed holocrine or apocrine modes of salivary secretion [240].

## Conclusion

Multiple tick salivary gland transcriptomes have predicted secreted tick salivary proteins [108, 114, 241–244], while two recent studies provided insight into the complexity of tick saliva proteomes [75, 200]. This study contributes to the emerging and growing knowledge on the complexity of the immunogenic *A. americanum* tick saliva proteome. Data here provides an interesting foundation on the range of candidate proteins to be screened in the quest to discover anti-tick target vaccine antigens. One of the key observations in this study is that housekeeping-like proteins were immunogenic, and thus must be given a new look in our search for anti-tick vaccine antigens. Given that housekeeping proteins tend to be highly conserved across taxa, one may assume that they were more likely not to provoke an immune reaction. Could it be that, although primary structures were highly conserved, there are important secondary structure departures that prevented the host from recognizing these proteins as self? The biopanning approach used here has limitations, and thus identification of some proteins here could be result of the cross-reactivity of antibodies used here with none-tick saliva proteins. We are confident that this is not the case in that previously confirmed tick saliva proteins, including tHRF [62], AV422 [70], paramyosin [236], defensin [235], selenoproteins [108], calreticulin [245], and histamine binding/lipocalins [54] were found in this study. This study though descriptive provides a foundation for the design of anti-tick target vaccine antigens.

### Availability of supporting data

All the supporting data are included as an additional file.

## Electronic supplementary material

Additional file 1: **List of sequences encoding hypothetical immunogenic 24-48 h fed** ***Amblyomma americanum*** **tick saliva proteins which do not match tick sequences present in GenBank.** (XLSX 25 KB)
